# Effects of a mixed berry beverage on cognitive functions and cardiometabolic risk markers; A randomized cross-over study in healthy older adults

**DOI:** 10.1371/journal.pone.0188173

**Published:** 2017-11-15

**Authors:** Anne Nilsson, Ilkka Salo, Merichel Plaza, Inger Björck

**Affiliations:** 1 Food for Health Science Centre, Lund University, Lund, Sweden; 2 Department of Food Technology, Engineering and Nutrition, Lund University, Lund, Sweden; 3 Department of Psychology, Lund University, Lund, Sweden; 4 Department of Chemistry, Centre for Analysis and Synthesis, Lund University, Lund, Sweden; Universita degli Studi di Milano, ITALY

## Abstract

**Background:**

Berries and associated bioactive compounds, e.g. polyphenols and dietary fibre (DF), may have beneficial implications with respect to the metabolic syndrome, including also cognitive functions. The aim of this study was to evaluate effects on cognitive functions and cardiometabolic risk markers of 5 wk intervention with a mixture of berries, in healthy humans.

**Methods:**

Forty healthy subjects between 50–70 years old were provided a berry beverage based on a mixture of berries (150g blueberries, 50g blackcurrant, 50g elderberry, 50g lingonberries, 50g strawberry, and 100g tomatoes) or a control beverage, daily during 5 weeks in a randomized crossover design. The control beverage (water based) was matched with respect to monosaccharides, pH, and volume. Cognitive tests included tests of working memory capacity, selective attention, and psychomotor reaction time. Cardiometabolic test variables investigated were blood pressure, fasting blood concentrations of glucose, insulin, blood lipids, inflammatory markers, and markers of oxidative stress.

**Results:**

The daily amounts of total polyphenols and DF from the berry beverage were 795 mg and 11g, respectively. There were no polyphenols or DF in the control beverage. The berry intervention reduced total- and LDL cholesterol compared to baseline (both P<0.05), and in comparison to the control beverage (P<0.005 and P<0.01, respectively). The control beverage increased glucose concentrations (P<0.01) and tended to increase insulin concentrations (P = 0.064) from base line, and increased insulin concentrations in comparison to the berry beverage (P<0.05). Subjects performed better in the working memory test after the berry beverage compared to after the control beverage (P<0.05). No significant effects on the other test variables were observed.

**Conclusions:**

The improvements in cardiometabolic risk markers and cognitive performance after the berry beverage suggest preventive potential of berries with respect to type 2 diabetes, cardiovascular disease, and associated cognitive decline. Possibly the polyphenols and DF contributed to the beneficial effects.

**Trial registration:**

ClinicalTrials.gov: NCT01562392.

## Introduction

Lifestyle habits play a pivotal role for development of obesity, type 2 diabetes (T2DM) and cardiovascular disease. It is increasingly recognized that T2DM potentiate the risk of cognitive decline, e.g. decline in working memory, verbal memory, executive functioning, information processing speed, attention, and increase the risk for dementia, including Alzheimer’s disease [1]. Also several of the individual key features that define the metabolic syndrome (MetS), e.g. hypertension, impaired glucose regulation, dyslipidemia, obesity [2], and inflammation [3], predispose for cognitive decline. In fact, accumulating data are in support of cognitive impairment being an early manifestation of the MetS, appearing even prior to impaired glucose tolerance [4, 5]. The diet is probably the most significant lifestyle factor of importance for the etiology of MetS, making the diet an essential target in a strategy aiming at preventing cardiovascular diseases, and probably also MetS and T2DM associated cognitive decline. With respect to cardiometabolic health, certain foods, e.g. whole grain foods [6], legumes [7], dietary fibre (DF) [8], and low glycaemic index (GI) foods [9], have been found to be advantageous. Furthermore, reports are available suggesting that foods and dietary patterns which induce cardiometabolic benefits also may improve cognitive functions [10]. Fruits and vegetables are rich sources of phytochemicals, e.g. polyphenols and carotenoids, and are good sources of DF. It has been reported that berry consumption has cardio protective effects. For example, a mix of berries (bilberries, lingonberries, blackcurrants, strawberries, chokeberries and raspberries) consumed for 8 weeks (wk) lowered blood pressure and improved blood lipid profile in middle aged subjects displaying cardiovascular risk factors [11]. Further, 8 wk blueberry supplement decreased markers of oxidative stress in obese men and women with the MetS [12] and improved insulin sensitivity [13] in obese subjects with insulin resistance. With respect to cognitive functions, prospective studies indicate that higher intake of berries such as blueberries and strawberries is associated with enhanced cognitive functions in elderly subjects [14]. However, information from intervention studies regarding effects on cardiometabolic risk markers and cognitive function of dietary supplementation with berries is still scarce.

The present study aimed to investigate effects of a mixture of berries (blueberries, elderberries, strawberries, blackcurrants, lingonberries, and tomatoes) on cognitive functions (working memory, selective attention and psychomotor reaction time) and on cardio-metabolic risk markers in healthy humans. Furthermore, an important part of this study implicated determinations of bioactive components and properties in the test products with respect to contents of polyphenols and antioxidant capacity, and with respect to the content of DF. The cardiometabolic test variables measured were; fasting concentrations of glucose and insulin, blood lipids (free fatty acids (FFA), triacylglycerol, total cholesterol (total-C), LDL cholesterol (LDL-C), and HDL cholesterol (HDL-C)), inflammatory markers (interleukin 6 (IL-6) and interleukin 18 (IL-18)), markers of oxidative stress (oxidized LDL-C (ox-LDL) and malondialdehyde (MDA)), and blood pressure. Forty healthy subjects between 50–70 years old and with BMI ≤ 28kg/m^2^ were provided the berry mixture daily for 5 wk in form of a beverage. In a randomized crossover study design the effects were compared to the effects of 5 wk intake of a control beverage, matched with respect to carbohydrate, pH, and volume.

## Material and methods

### Ethical statement and trial registration

This study was conducted in compliance with the guidelines laid down in the Declaration of Helsinki (ethical principles for research involving human subjects). Written informed consent was obtained from all subjects. All procedures involving human subjects were approved (September 9, 2011) by the Regional Ethical Review Board in Lund, Sweden (Dnr 2010/457 and 2011/510). The study started in September 2011. The recruitment was finished August 2012, and the study was completed January 2013. The study was registered Mars 15, 2012 at ClinicalTrials.gov: NCT01562392. The delay in the registration of the study was due to an administrative error due to the human factor. The authors confirm that all ongoing and related trials for this intervention are registered. The supporting CONSORT checklist (S1 Checklist) and the trial protocol for this study (S1–S3 Text) are available as supporting information.

### Study population

The inclusion criteria were apparently healthy non-smoker volunteers with an age of 50–70 years old and a normal to slightly increased body mass indices (BMI) (≤ 28 kg/m^2^). The exclusion criteria were fasting blood glucose > 6.1 mmol/L, known metabolic disorders, food allergies, gastro- intestine disorder or known cognitive disorders which could affect the results. Due to the construction of the cognitive tests the subjects should be fluent in the Swedish language.

**Fig 1:**
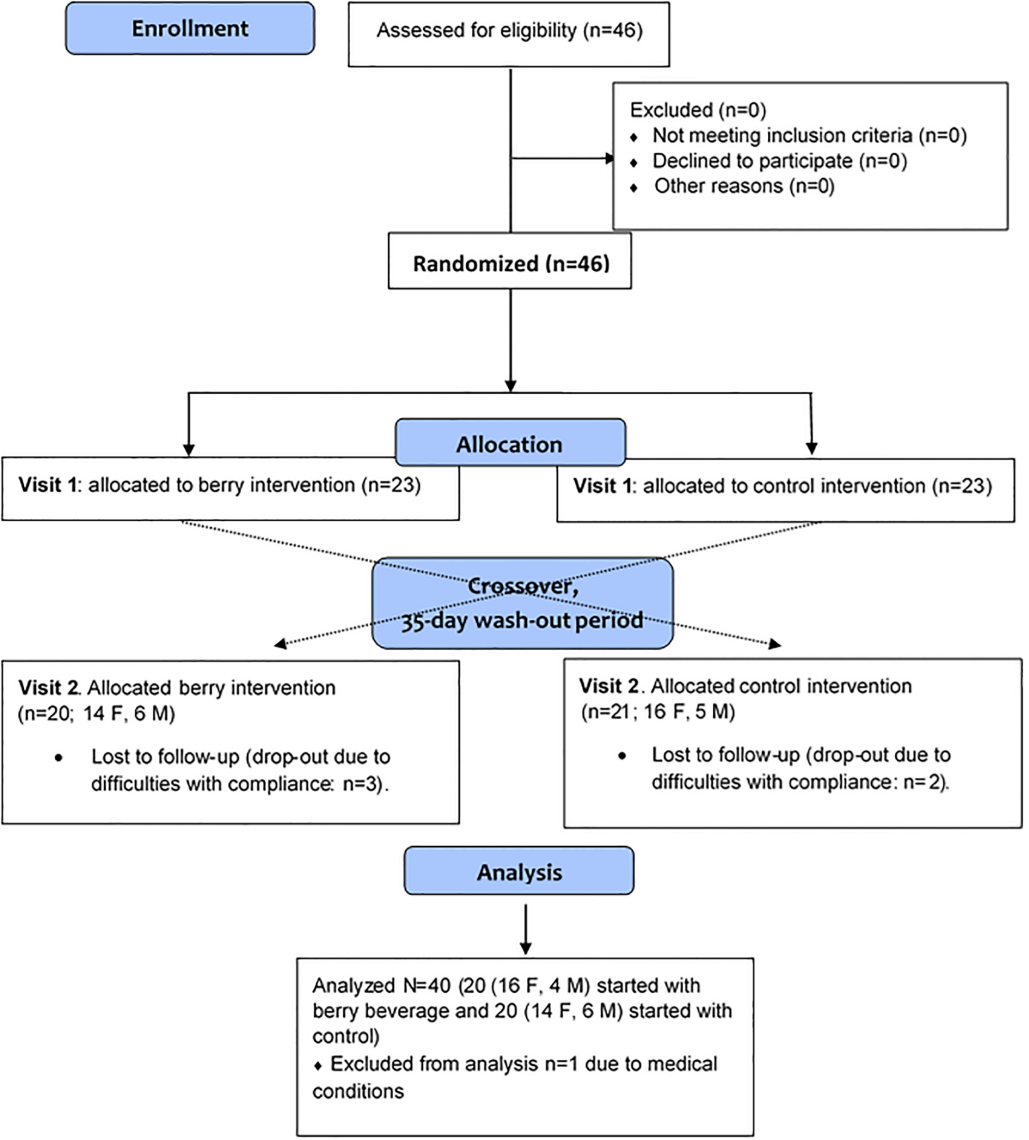
CONSORT flow diagram of the study progress.

The recruitment started in September 2011, and the experimental work was completed in January 2013. Forty-six healthy men and women from the south of Sweden were recruited to the study. Forty-one subjects completed the study, however, one subjects were excluded after completion due to medical reasons. Accordingly, forty subjects were included in the statistical evaluations (see Result section for more comprehensive information). A flow diagram of the study progress is displayed in Fig 1.

### Study design and protocol

The study had a cross-over randomized but balanced experimental design. Of the 40 subjects that were included in the statistical evaluation, twenty subjects started with five wk daily consumption of the berry beverage and consumed the control beverage in a second five wk intervention period (BC group), and 20 subjects started with five wk consumption of the control and had the berry beverage in the last five wk period (CB group). The intervention periods were separated by a five wk washout period. The subjects were instructed to avoid dark and colorful berries and berry products during the interventions, and to avoid alcohol, food rich in DF, and excessive physical exercise the day prior to the experimental days. No antibiotics or probiotics were allowed within two weeks before or during the study period.

All subjects participated at four experimental days, these were: the day prior to the start of each intervention period (i.e. the day before the start of the control intervention (C_0_) and the day before the start of the berry intervention (B_0_)), and the day after the completion of each intervention period (i.e. the day after 5 wk consumption of berry beverage (B_5wk_) and the day after 5 wk consumption of control beverage (C_5wk_). The experiments were executed at the Food for Health Science Center at Lund University, Sweden. The evening prior to attendance, at 9.00 pm. the test subjects consumed a standardized meal consisting of white wheat bread with optional spread, and had coffee, tea or water to drink. When an experimental day was preceded by an intervention period (test occasion 2 and 4), a last portion (200 ml) of the test- or control product was consumed together with the late evening meal at 9.00 pm. After the evening meal the subjects were fasting until the arrival at the research unit the following morning (07.30 am). After arrival, the test subjects were weighed and seated to rest for a minimum of 10 minutes before registering the blood pressure and withdrawing fasting blood tests for determination of physiological test parameters (see below). Thereafter a standardized breakfast was provided, consisting of 87 g white wheat bread (Hennings storfranska, 600 g, Fazer Bakery inc.), 25 g apricot marmalade (ICA, Sweden), 23 g cheese (Gouda cheese, ICA Basic 400g, ICA, Sweden), 150 ml decaffeinated coffee or tea (individual standardized) without sugar and milk, and 100 ml water. The standardized breakfast provided in total 50 g available carbohydrates. The breakfast was consumed within 15 min. Cognitive tests (see below) were performed repeatedly in the postprandial period at test occasions 2 and 4, i.e. after each intervention period. At test occasions 1 and 3, i.e. at start of the intervention periods, the subjects performed pilot versions of the cognitive tests to reduce learning effects and stress at the cognitive test days. The test subjects were told to maintain a low physical activity during the 3 hours of repeated sampling of test variables.

### Test- and control products

Test product: The test product was consumed in the form of a beverage produced specific for this study (by Orkla Foods Sverige AB, Malmö, Sweden) and based on a mixture of Swedish berries, selected on the basis of being known to be rich in polyphenols or carotenoids. The daily portion berry mixture was based on 150 g blueberry, 50 g elderberry, 50 g lingonberry, 50 g strawberry, 50 g blackcurrant, and an amount of tomato powder (6g) corresponding to approximately 100 g fresh tomatoes. The frozen and then thawed mix of berries was diluted with water, 40%, and pressed through a 2 mm filter. A small amount (1%) sugar was added to the beverage. After pasteurization (95°C, 30 sec) the beverage was packaged in portions of 200 ml (~203g). The total amount of berry beverage per day was 600 ml (~609 g), equal to three packages, supposed to be consumed with the meals at breakfast, lunch and dinner. The test- and control beverages were characterised with respect to macronutrient composition, DF (insoluble and soluble), pH, polyphenols and antioxidant capacity (see below) (Table 1).

**Table 1:** Characterization of the berry- and control beverages, respectively, with respect to macro nutrients, DF (insoluble and soluble) and pH.

	Berry beverage	Control beverage
Glucose (%)^1^	2.2	2.2
Fructose (%)^1^	3.4	3.4
Sucrose (%)^1^	<0.1	0
Protein (%)^1^	0.6	0
Fat (%)^1^	0.3	0
Insoluble DF (%)	1.35	0
Soluble DF (%)	0.45	0
pH	3.2	4.0

^1^Analyzed by ALcontrol AB, 212 39 Malmö, Sweden.

Control product: The control product (produced specific for this study by Source Food Production AB, Hammargränd 2, SE-275 39 Sjöbo, Sweden) was composed of a beverage matching the test product with respect to type and amount of low-molecular weight carbohydrates (sucrose, glucose, and fructose) and pH (adjusted with citric acid) (Table 1). Alike the test product, the control beverage was packed in aliquots of 200 ml (200 g) to be consumed with the meals at breakfast, lunch, and dinner (600 ml/day).

### Analysis of polyphenols and determination of antioxidant capacity in the berry- and control beverages

The phenolic compounds in the berry- and the control beverages were analyzed with HPLC with hyphenated diode array, electrochemical and charged aerosol detection (HPLC-DAD-ECD-CAD system), and the antioxidant capacity was measured using Trolox equivalent antioxidant capacity (TEAC) assay and Folin-Ciocalteu reducing capacity (FC method) *in vitro* assays (see below). The FC assay is one of the oldest methods developed to determine the content of phenols [15]. FC reagent is nonspecific to phenolic compounds, and for that reason, it should be considered not an accurate method for determination of total phenolic content unless interfering species are consider or removed [16]. Therefore, the FC assay can be used for the measurement of total reducing capacity. Both methods were used in this study to take into account two different antioxidative mechanisms present in the complex berry beverage.

#### Sample preparation for analysis of phenolic compounds and antioxidant capacity determination

The berry beverage (4 packages) and the control product (1 package) were centrifuged during 10 min. The supernatant was then filtrated through Teflon filter (0.2 μm). The sample was protected from light, and storage at -20°C until analysis.

#### Evaluation of antioxidant capacity

**TEAC assay:** The TEAC assay described by Re et al. [17] with some modifications was used to measure the antioxidant capacity of the berry beverage and the control product. ABTS radical cation (ABTS^**·**+^) was produced by reacting 7 mM ABTS with 2.45 mM potassium persulfate and allowing the mixture to stand in the dark at room temperature for 12–16 h before use. The aqueous ABTS^**·**+^ solution was diluted with 5 mM phosphate buffer (pH = 7.4) to an absorbance of 0.70 (± 0.02) at 734 nm. Ten microliters of beverage (four different dilutions) was added to 1 ml of diluted ABTS^**·**+^ radical solution. After 50 min at 30°C, 300 μl of the mixture were transferred into a well of the microplate, and the absorbance was measured at 734 nm in a microplate spectrophotometer reader (Multiskan GO, Thermo Fisher, Germering, Germany). Trolox was used as a reference standard and results were expressed as TEAC values (mmol Trolox/l of beverage). These values were obtained from at least three different concentrations of each beverage (4 berry beverage packages and 1 control product) tested in the assay giving a linear response between 20–80% of the initial absorbance. All analyses were done in triplicate.

**FC Assay:** The Folin-Ciocalteu reducing capacity was estimated as gallic acid equivalents (GAE), expressed as mg gallic acid/l of beverage [18]. The total volume of the reaction mixture was miniaturized to 1 ml. 10 μl of sample were mixed, to which 50 μl of undiluted Folin-Ciocalteu reagent was subsequently added. After 1 min, 150 μl of 2% (w/v) Na_2_CO_3_ and 790 μl of water were added. After 2 h of incubation at 25°C, 300 μl of the mixture was transferred into a well of the microplate, the absorbance was measured at 760 nm in a microplate spectrophotometer reader (Thermo Scientific) and compared to a gallic acid calibration curve (0.025–2.000 mg/ml) elaborated in the same manner. The data were presented as the average of triplicate analyses for each product.

#### Analysis of polyphenols with HPLC-DAD-ECD-CAD

The phenolic compounds of the berry beverage and the control product were analyzed with a HPLC-DAD-ECD-CAD system according to previous work with some modifications [19, 20]. Separation was carried out with porous-shell fused core Ascentis Express C18 analytical column (150 mm x 2.1 mm, 2.7 μm) from Supelco (Bellefonte, PA, USA). The mobile phases consisted of (A) 60 mM ammonium formate buffer (pH 1.5) in water, and (B) methanol with 5% of formic acid in a gradient elution analysis programmed as follows: 0 min, 5% (B); 0–5 min, 5% (B); 5–35 min, 40% (B); 35–40 min, 40% (B); with 10 min of post-time for column conditioning at a flow rate of 300 μl/min. It has been shown that the CAD response strongly depends on the amount of organic solvent in mobile phase [19]. In order to achieve a uniform CAD response and to be able to quantify the phenolic compounds with just one phenolic standard an inverse methanol gradient (make-up gradient) requiring a dual gradient pump system was needed [21, 22]. The make-up gradient started 0.3 min after the elution gradient. All solvents were purged continuously with nitrogen to remove oxygen. The column temperature was set at 50°C, the injection volume was 2 μl and the vial tray was held at 4°C.

Phenolic compounds were quantified with CAD detector. The calibration curve of cyanidin 3-glucoside was selected to quantify all phenolic compounds. The cyanidin 3-glucoside standard solution was injected by triplicate at six concentrations levels (1–100 μg/ml). The calibration curve of cyanidin 3-glucoside was obtained by plotting peak area as function of concentration (μg/ml). Responses obtained in the examined ranges were expressed by a linear equation, y = ax ± b (y = 3.3017x − 0.0023), with good r^2^ determination coefficient value (0.997).

### Cognitive- and physiological test variables

#### Cognitive tests

Determinations of cognitive performance were performed after completion of each intervention period, i.e. at visit no. 2 and 4.

**Verbal working memory (WM) test:** The test was as originally described by Daneman and Carpenter [23]. However the tests employed in the present study represent an extension of the methodology, developed by Radeborg et al. [24]. WM can be defined as a system responsible for simultaneous temporary short term storing and processing of information, and is involved in many everyday activities; such as mathematical problem solving or reasoning where one often has to remember one part while performing further operations. WM represent a fundamental ability for higher-level cognitive processes. Some authors [25, 26] even claim that WM and general problem solving ability or intelligence, as measured by e.g. Raven´s Matrices, reflect nearly identical constructs. However, whereas intelligence tests generally only can be administered once due to risk of considerable learning effects, WM can be measured repeatedly. The tests consisted of 12 sets of 3–5 short declarative sentences that could be either semantically meaningful of the type ‘the boy brushed his teeth’, or nonsensical, such as ‘the rabbit struck the idea’. Each test has equal number of sets with 3, 4, and 5 sentences (4 of each). The sentences were read one by one to the subjects, and immediately after each sentence the subject had to indicate if the sentence was semantically meaningful or not. The subjects were blind to the number of sentences in each set. After each set of sentences, the subjects had to repeat, in any order, the first noun in each of the sentences. Six different but comparable WM-tests were included in the study, and three tests were performed at each cognitive test day, executed at 30, 90, and 150 min after commencing the standardized breakfast. The first WM-test at a test day took approximately 10 min to perform, included short information and training session, and the second and third test occasions took approximately 6 min each to perform. One test could at maximum generate 48 credits. The tests consisted of equal number of sentences that were semantically meaningful (24 credits) and nonsensical (24 credits).

**Selective attention (SA) test:** The test was based on spatial perception and primarily measured the ability to sustain a prolonged attention, and to control and split the attention to the entire picture on a computer screen. Like the WM-test, the test also dealt with simultaneous temporary storing and processing of information (WM capacity). The storing time required was however shorter compared with the WM-test, whereas the time pressure was higher. In addition, the test measured psychomotor speed. The SA was measured using a computerized test made up of 96 pictures, each shown for two seconds on the computer screen. The pictures consisted of a square on a white background, divided into four equally sized smaller squares. One of the smaller squares was red, one square was green, and two squares were uncolored (white), resulting in a total of 12 unique picture combinations. The subjects had to remember the positions of the colored squares, and to compare each new picture that emerged on the screen with the preceding one. Each time a new picture emerged, either the green, the red, or none of the colored squares were in the same position compared with the previous picture. Within the two seconds each picture was shown, the subjects were supposed to as fast as possible indicate by pressing one of three different keys on the keyboard, which of the three possible alternatives that occurred for each new picture. The test began with a short training session, and took approximately 8 min to perform. The test was scored with the number of correct responses (CR, total 95 credits) and for the reaction time (RT) needed to give the answer (i.e. press one of the keys). Two SA tests were included at each cognitive experimental day, performed at 60 and 120 min after commencing the standardized breakfast.

#### Physiological test variables

Physiological test variables were determined at fasting at all four visits, i.e. prior to and after completing each intervention period. Blood pressure was determined with an automatic blood pressure cuff (Digital Automatic Blood Pressure Monitor, Model M3 Intelligence, OMRON HEALTHCARE CO., LTD, Kyoto, Japan). Finger-prick capillary blood was withdrawn for determination of glucose concentrations (HemoCue^®^B-glucose, HemoCue AB, Ängelholm, Sweden). Venous blood was withdrawn for determination of serum (s) insulin, s-FFA, s-triacylglycerol, s-IL-6, s-IL-18, s-total-C, s-LDL-C, s-HDL-C, s-ox-LDL), and plasma (p) MDA. The venous blood samples were centrifuged and plasma and serum separated and stored in a freezer (−40°C) until analyzed.

*S-insulin* was determined with a solid phase two-site enzyme immunoassay kit (Insulin ELISA 10-1113-01, Mercodia AB, Uppsala, Sweden, and *s-FFA* concentrations with an enzymatic colorimetric method using a 96 well microplate (NEFA C, ACS-ACOD method, WAKO Chemicals GMbH, Germany). The quantitative determination of *s-IL-6* was performed with an enzyme immunoassay (Human IL-6 HS600B, R&D Systems, Abingdon, UK) and *s-IL-18* with an enzyme immunoassay that was modified in the sense that no dilution of serum was performed prior to the analysis (Human IL-18 ELISA Kit 7620, MBL Medical & Biological Laboratories CO., Ltd, Nagoya, Japan). *S-ox-LDL-cholesterol* was quantified with an enzyme-linked immunosorbent assay (ox-LDL/MDA Adduct ELISA Kit, Immundiagnostik AG, Bensheim, Germany), and *p-MDA* was determined by measure of lipid peroxidation as TBARS as is described in [27], modified by excluding the n-butanol. *S-triacylglycerol* was analyzed with a multi-sample enzymatic assay (LabAssay^™^ Triglyceride art.nr 290–63701, GPO·DAOS method, Wako Chemicals GmbH, Neuss, Germany), *s-HDL-C and s-LDL-C* were assayed with an enzymatic selective protection method Kit (WAKO Chemicals GMbH, Germany). *Total-C* was calculated from the results of HDL-C, LDL-C and triacylglycerol using Friedewald’s equation [28]. HOMA-IR was calculated from fasting blood plasma-glucose and serum insulin values [29].

### Calculations and statistical methods

Cognitive assessments were obtained at the test occasions after completion of each intervention period, i.e. at visits no. 2 and 4. Cardiometabolic test markers were obtained both prior to start of each intervention period, i.e. at visits no. 1 and 3, and after the completion of each intervention period (visits no. 2 and 4). Primary outcome measure was results in the WM-test. The sample size was calculated based on a study in healthy middle aged subject, including a similar WM-test as was used in the current study [24]. A significant effect (*p* < 0.05) was detected on WM, with an effect size (Cohen´s d) of *d* = 0.75. In the present study we assumed a smaller effect (*d* = 0.50), resulting in a power of 0.77 (two tailed test). In a power calculation, based on table 9–9 and 9–10 in Aron and Aron 82003: Statistics for Psychology, 33 subjects would be enough to get a 80% power (two tailed test). However, assuming a normal drop-out frequency we decided to involve 46 subjects. Included in the statistical calculations were 40 subjects. In the SA-test (n = 39) one test subject was excluded due to improper execution of the test. Randomization of the treatment sequence (i.e. berry at the first intervention period and control in the second period (BC group, n = 20), or control at the first period and berry in the second period (CB group, n = 20)) was performed using a random number function in Microsoft Excel 2013 (Washington, USA).

The statistical evaluation of reaction time in the SA-test is based on median reaction times for correct answers at respective test points. The influences of the test- and control products on the cognitive tests were analyzed by repeated measures ANOVA (two-tailed tests) at the test points, with treatment and time (i.e. test points during the test days) as independent variables, and performance on cognitive tests as dependent variables. Statistical calculations were performed in Stat View 5.0 and SuperAnova 1.11. Investigations of differences in performance in the cognitive tests at each test point, and effects on cognitive performance of treatment sequence (BC or CB sequence), time during the test day, and interactions were in addition assessed with analysis of variance (one-way ANOVA, two-tailed test, general linear model (GLM)) in MINITAB (release 17; Minitab, Minitab Inc, State College, PA). Treatment effects of berry beverage (B) (B_5wk_ compared with B_0wk_, n = 40) and control beverage (C) (C_5wk_ compared with C_0wk_, n = 40), respectively, on physiological test parameters, and differences between treatments (B compared with C), as well as effects of treatment sequence and interactions were assessed with ANOVA GLM, in MINITAB (two-tailed test). Standard error of the mean (SEM) is used to present how precisely the sample mean estimates the population mean, thus the results are expressed as means ± SEM. The study design was cross-over, allowing for the participants to act as their own control. The significance level was set at *P*-values ≤ 0.05.

## Results

### Study population

Five subjects dropped out after the first visit due to difficulties with compliance (three in the BC-group and two in the CB group). One subject in the CB group was excluded after completion of the study due to supplementary information concerning medical condition. Consequently, out of the 46 subjects recruited to the study, 40 subjects (30 women and 10 men) completed the study. Baseline data collected at the time of the first clinical visit for the 40 subjects that were included in the statistical evaluations are shown in Table 2.

**Table 2:** Characteristics of test subjects at baseline.

	Whole group (n = 40)	Women (n = 30)	Men (n = 10)
Age (y)	63.0±0.9	63.1±1.0	62.7±2.0
Body weight (kg)	71.1±1.7	68.5±1.7	79.0±3.2
BMI kg/m^2^	24.4±0.4	24.5±0.5	24.1±0.7
Blood pressure (mmHg)			
Systolic	133.0±2.8	129.2±3.0	144.3±5.0
Diastolic	86.0±1.7	84.9±2.0	89.3±2.9
Glucose (mM)	5.2±0.07	5.1±0.08	5.4±0.2
Insulin (pM)	34.7±3.1	32.7±2.3	40.5±9.8
HOMA-IR	1.4±0.1	1.2±0.1	1.7±0.4
Cholesterol (mM)			
Total-C	5.7±0.1	5.8±0.1	5.4±0.3
HDL-C	1.2±0.1	1.3±0.1	1.2±0.1
LDL-C	3.8±0.1	3.9±0.2	3.7±0.3
Triacylglycerol (mM)	1.4±0.1	1.3±0.1	1.7±0.2
FFA (mM)	0.34±0.02	0.36±0.02	0.31±0.02
IL-6 (ng/L)	1.4±0.2	1.3±0.2	1.7±0.6
IL-18 (ng/L)	276.9±15.1	277.5±18.9	274.8±22.5
MDA (μM)	2.4±0.1	2.3±0.1	2.5±0.1
ox-LDL (pg/L)	128.0±17.0	125.3±19.8	136.4±35.2

According to the definition as defined by IDF (The International Diabetes Federation) (central obesity (or BMI > 30 kg/m^2^), triglycerides > 1.7 mmol/L, HDL cholesterol < 1.03 mmol/L in males and < 1.29 mmol/L in females, systolic BP > 130 or diastolic BP > 85 mm Hg (or treatment of diagnosed hypertension), and fasting plasma glucose > 5.6 mmol/L), one of the female subjects met the criteria for the MetS (BMI 31 kg/m^2^, HDL-cholesterol 0.6 mmol/L, and triglycerides 2.27 mmol/L). Only seven subjects (17.5%) out of the 40 subjects exhibited none of the MetS, as defined by IDF. One male subject had fasting glucose concentration above 6.1 mmol/L (6.3 mmol/L) at the first visit, however the fasting concentrations decreased to < 6.1 mmol/L at the rest of the visits. Applying the WHO definition of the MetS symptoms, (i.e. diabetes mellitus, impaired glucose tolerance, impaired fasting glucose (glucose > 6.1 mmol/L) or insulin resistance, and two of the following: blood pressure: ≥ 140/90 mmHg, dyslipidemia: triglycerides ≥ 1.695 mmol/L and HDL-C ≤ 0.9 mmol/L in males and ≤ 1.0 mmol/L in females, central obesity (waist:hip ratio > 0.90 (male); > 0.85 (female)) or body mass index > 30 kg/m^2^, urinary albumin excretion ratio ≥ 20 μg/min or albumin:creatinine ratio ≥ 30 mg/g), 22 subjects in the study population (55%) exhibited none of the MetS symptoms, and none of the subjects met the criteria for the MetS. It must however be noted that albumin excretion was not investigated in the present study.

No specific screening for possible cognitive decline was carried out prior to the enrollment; however, performance in the pilot versions of the tests at visit 1 was taken as a measure of the subjects' cognitive abilities to conduct the study in an adequate manner. All participants were considered qualified to participate in the study.

### Polyphenols and antioxidant capacity in the berry- and control beverages

**Fig 2:**
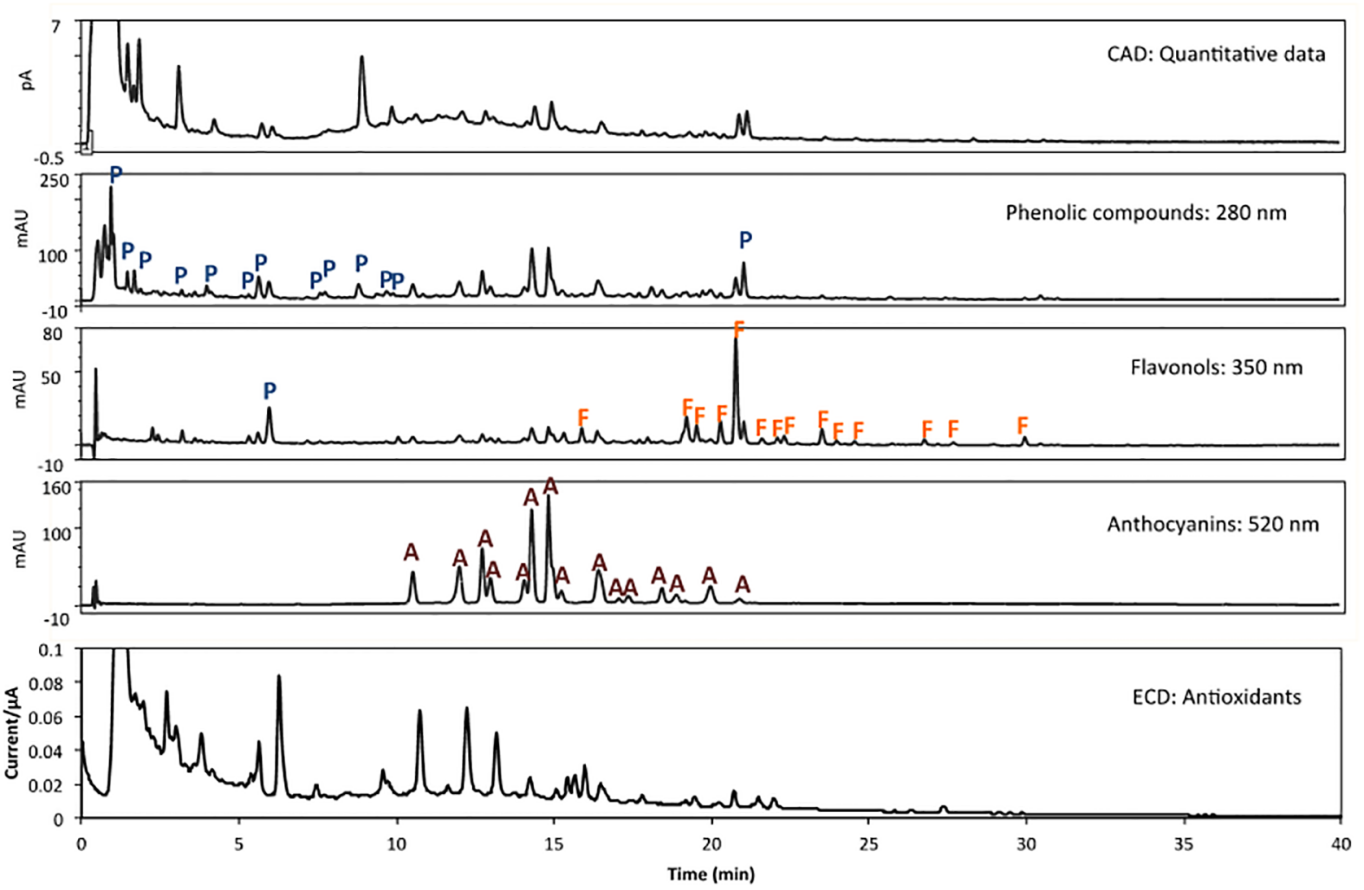
Chromatograms and amperograms corresponding to the HPLC-DAD-ECD-CAD analysis of berry beverage at 280 nm, 350 nm and 520 nm and amperogram. P, phenolic compounds; F, flavonols; and A, anthocyanins.

In order to separate the phenolic compounds by family group, measure the antioxidant power, and quantify the phenolic composition of the berry- and control beverages, a HPLC-DAD-ECD-CAD method was set up. The chromatograms obtained (Fig 2) clearly demonstrated that several phenolic compounds could be separated in less than 30 min. The analysis of the separated compounds, using the information provided by the DAD, allowed the identification of the phenolic compound for family groups. For instance, the selected wavelengths of 280, 350 and 520 nm made possible the identification of phenolic compounds, flavonols and anthocyanins, respectively. Fifteen anthocyanins, 14 flavonols and 14 phenolic compounds (phenolic acids and flavanols among others) were found in the berry beverage. The control beverage did not present phenolic compounds. CAD detector was used to carry out the quantification of the phenolic compounds found in berry beverage. As shown in Table 3, the total phenolic concentration in the berry beverage was 1324.9 mg/l. Anthocyanins represented a phenolic group found in high concentration in the berry beverage (414.2 mg/l). Other groups of compounds found in considerable concentration in the berry beverage were the flavonols (155.9 mg of flavonols/l).

**Table 3:** Characterization of the berry- and control beverages with respect to total polyphenols, anthocyanins, flavonols, and antioxidant capacity.

	Berry beverage	Control beverage
Total polyphenols (mg/l)	1324.9±103.2	0
Anthocyanins (mg/l)	414.2±32.8	0
Flavonols (mg/l)	155.9±8.7	0
*Antioxidant contribution of phenolics (ECD peak area)*:		
Total (μA*s)	4.9±0.1	0
Anthocyanins (μA*s)	2.1±0.1	0
Flavonols (μA*s)	0.6±0.03	0
*Total antioxidant capacity*:		
Folin method (mgGAE/l)	805.7±27.9	0
TEAC method (mmol trolox/l)	20.3±1.6	0

ECD was used to assess the antioxidative properties of the phenolic compounds present in the berry beverage (Fig 2, Table 3). The anthocyanins represented 31% of the total phenolic compounds, but their contribution to the total antioxidant capacity of the phenolics was 43%. This means that the contribution of anthocyanins to the total antioxidant capacity was considerable. The flavonols represented 12% of the total phenolic compounds and their contribution to the total antioxidant capacity of the phenolic compounds was approximately the same (11%). Both groups (anthocyanins and flavonols) represented more than the half of the antioxidant capacity from phenolic compounds (54%). The rest of phenolic compounds from other groups (phenolic acids, flavanols, ellagic acid, among others) represented 57% of the total phenolic compounds, but their contribution to the total antioxidant capacity was lower (46%).

The berry beverage was able to act against ABTS^•+^ and FC reagent. The antioxidant capacity of the berry beverage in this study was 20.3 mmol trolox/l determined by the TEAC method, and 805.7 mg of GAE/l measured with the FC method (Table 3). As expected, the control beverage did not show antioxidant capacity.

### Cognitive performance

The results from the WM-tests after 5 wk intervention with the berry beverage and the control beverage, respectively, are presented in Table 4. No main effect depending on treatment (F(1, 38) = 1.75, *P* = 0.194) or treatment * time interactions (F(1, 38) = 1.31, *P* = 0.283) were observed in the WM-test when all test points were included in the statistical calculations (two way repeated measures ANOVA). On the contrary the results in the repeated measures ANOVA revealed significant main effects of time during the experimental days, F(2, 37) = 6.08, *P* = 0.005, showing inferior performance at the last WM-test of the test days, i.e. the test performed at 150 min after start of the standardized breakfast (30.6±0.7 credits), compared to performance at 30 min (31.5±0.6) and 90 min (31.8±0.6 credits), respectively.

**Table 4:** Results in the working memory tests after five wk interventions with daily intake of the berry beverage and control beverage, respectively^1^.

WM-test (n = 40) (max 48 credits)	Treatments	
	Berry beverage	Control beverage
30 min	32.5±0.7^a^	31.0±0.9 ^b^
90 min	31.7±0.8 ^a^	31.3±0.8 ^a^
150 min	30.4±0.7 ^a^	30.7±0.9 ^a^

^1^Data are given as means ± SEM.

Values in the same row with different superscript letters (a, b) are significantly different (at 30 min: F(1, 39) = 4.55, *P* = 0.039, one-way ANOVA GLM).

There were no differences in the performance in the WM-tests depending on the treatment sequence of the intervention products, i.e. between BC group and CB-group (mean word retrieval in WM-tests 1–3: F(1, 38) = 1.63, *P* = 0.209, one-way ANOVA GLM). However, there was a trend towards a [treatment*treatment sequence] interaction in the WM-test (mean WM-tests 1–3, F(1, 38) = 4.06, *P* = 0.051, one-way ANOVA GLM), indicating better performance after the berry beverage compared with the control in the CB group (F(1, 18) = 6.32, *P* = 0.022, one-way ANOVA GLM), whereas there were no significant differences depending on treatment in the subjects that had the opposite treatment sequence, i.e. in the BC group (F(1, 19) = 0.17, *P* = 0.689, one-way ANOVA GLM). The significant time effects and trends towards treatment*treatment sequence interactions make it relevant to investigate performance in the WM-test at the different time points. The results show that five week intervention with the berry beverage improved performance in the WM-test at 30 min after the standardized breakfast by approximately 5% in comparison to the control beverage (F(1, 39) = 4.55, *P* = 0.039). The improvements at 30 min was most pronounced in the CB group (F(1, 19) = 9.43, *P* = 0.006, one-way ANOVA GLM).

The interaction showing significant improvements from the first cognitive test day to the second test day only when berry beverage was consumed in the last intervention period, and the absence of improvement with time during the test days, indicate that there were no significant learning effects in the WM-test.

No significant main effects were observed in the SA-test with respect to correct responses (F(1, 38) = 0.04, *P* = 0.948) or reaction time (F(1, 38) = 0.1, *P* = 0.760) depending on test products (two way repeated measures ANOVA, Table 5). Alike the WM-test there were significant time effects during the experimental days in both correct responses (F(1, 38) = 24.6, *P <* 0.001) and reaction times (F(1, 38) = 43.2, *P <* 0.001) (repeated measures ANOVA). However, in the opposite to the WM-tests, the results in the SA-tests revealed superior performance at the last test point during the test days compared to the first test point (64.6±2.0 credits, 1317±20 ms and 70.0±1.8 credits, 1237±21 ms, for 60 min and 120 min, respectively). No significant differences were detected in the performance in the SA-tests depending on the treatment sequence of the intervention products, i.e. starting with berry beverage (BC group) or starting with the control beverage (CB group) (mean score and reaction time in the SA-tests 1–2: F(1, 37) = 0.00, *P* = 0.949 and (F(1, 37) = 0.70, *P* = 0.409), respectively). However, there was pronounced [treatment* treatment sequence] interactions, in the SA-tests, both in scores (F(1, 38) = 29.3, *P <* 0.001) and in reaction time (F(1, 38) = 20.3 *P <* 0.001). The interaction displayed a better performance after the second intervention period independently of product consumed. The results in the SA-tests thus indicated pronounced learning effects.

**Table 5:** Results in selective attention tests before and after five wk interventions with daily intake of the berry beverage and control beverage, respectively^1^.

SA-test (n = 39) (max 96 credits)	Treatment	
	Berry drink	Control
*60 min*		
Correct responses	64.1±3.0	65.1±2.8
Reaction time (s)	1.3±0.02	1.3±0.02
*120 min*		
Correct responses	71.3±2.6	70.5±2.6
Reaction time (s)	1.2±0.02	1.2±0.02

^1^Data are given as means per treatment ± SEM.

### Cardiometabolic risk markers

The results concerning effects of 5 wk interventions with the berry- and the control beverage, respectively, on cardiometabolic risk markers are displayed in Table 6. There were no significant differences between baseline values prior to start of the berry intervention (i.e. B_0_) compared with prior to start of the control intervention (i.e. C_0_) for any of the test variables (Total-C: *P* = 0.198, HDL: *P* = 0.541, FFA: *P* = 0.420, MDA: *P* = 0.491, and the rest of the test markers *P* > 0.840). Neither were there any interactions between treatment sequence (BC and CB) and differences in changes from baseline (C_0_ and B_0_, respectively) depending on treatments (C_5wk_ and B_5wk_, respectively) (*P* = 0.136 for IL-6, *P* = 1.91 for glucose, and for the rest of test variables *P* > 0.269). The berry beverage (B_5wk_) lowered the total-C (-3.4%, F(1, 36) = 5.2, *P* = 0.029) and LDL-C (-4.6%, F(1, 39) = 7.1, *P* = 0.011) concentrations compared to baseline concentrations (B_0_). The total-C and LDL-C concentrations after the berry intervention was improved also in comparison with the effects of 5 wk treatment with the control beverage: F(1, 36) = 9.6, *P* = 0.004, and F(1, 39) = 8.5, *P* = 0.006, for differences in effects on total-C and LDL-C, respectively. No significant effects were observed on fasting glucose concentrations after berry intervention compared with B_0_. The opposite was observed after the control beverage, accordingly, fasting glucose concentrations were significantly increased (3.4%) after the control intervention compared with C_0_ (F(1, 38) = 8.3, *P* = 0.007). The berry intervention resulted in a non-significant reduction of the fasting insulin concentrations (-5.8%, *P* = 0.253) compared to B_0_, whereas the control intervention instead resulted in a tendency towards increased insulin concentrations by 12.8% compared to C_0_ (F(1, 37) = 3.7, *P* = 0.063). In consequence, the fasting insulin concentrations after the control intervention was significantly increased from baseline in comparison with the effects of the berry intervention (F(1, 37) = 4.3, *P* = 0.046). In addition the HOMA-IR increased from C_0_ after the control intervention (F(1, 36) = 5.3, *P* = 0.027). The changes from C_0_ was significantly increased after the control beverage in comparison to changes from B_0_ after the berry beverage F(1, 36) = 10.4, *P* = 0.003). No significant differences were observed concerning the other physiological test variables.

**Table 6:** Body weight, blood pressure and metabolic parameters in blood at fasting, prior to and after five wk interventions with the berry beverage and control beverage, respectively^1^.

	Treatments						
	Berry beverage			Control beverage			
Test variables	B_0_^2^	B_5wk_^3^	Change (%)^4^	C_0_^5^	C_5wk_^6^	Change (%)^7^	*P* (Δ berry- Δcontrol)^8^
Weight (kg, n = 40)	71.4±1.7	71.4±1.7	0.0	71.3±1.7	71.5±1.7	0.3	0.368
Systolic BP (mmHg, n = 40)	130.3±3.3	127.1±2.9	-2.4	129.7±2.9	126.7±2.7	-2.3	0.935
Diastolic BP (mmHg, n = 40)	84.1±1.8	82.3±1.7	-2.2	85.1±1.6	83.0±1.5	-2.4	0.877
Glucose (mM, n = 39)	5.2±0.1	5.3±0.1	1.3	5.3±0.1	5.4±0.1	3.4 **	0.186
Insulin (pM, n = 38)	34.4±2.5	32.4±2.7	-5.8	34.7±3.1	39.2±3.6	12.8 ^9^	0.046
HOMA-IR (n = 37)	1.3±0.1	1.3±0.1	-3.8	1.4±0.1	1.6±0.2	16.8 *	0.003
FFA (mM, n = 38)	0.3±0.01	0.3±0.02	1.8	0.3±0.02	0.3±0.02	-1.8	0.646
Total-C (mM, n = 37)	5.8±0.1	5.6±0.1	-3.4 *	5.6±0.1	5.8±0.1	3.4	0.005
LDL-C (mM, n = 40)	3.9±0.1	3.7±0.1	-4.6 *	3.8±0.1	3.9±0.1	2.4	0.006
HDL-C (mM, n = 37)	1.2±0.1	1.2±0.1	0.0	1.2±0.1	1.2±0.1	1.8	0.759
Triacylglycerol (mM, n = 39)	1.4±0.1	1.4±0.1	3.0	1.4±0.1	1.4±0.1	6.1	0.486
Total-C/HDL-C (n = 37)	5.3±0.4	5.0±0.3	-6.2	5.2±0.3	5.3±0.3	1.1	0.357
LDL-C/HDL-C (n = 37)	3.7±0.4	3.4±0.2	-8.2	3.7±0.3	3.7±0.3	0.5	0.357
IL-6 (ng/L, n = 39)	1.2±0.2	1.2±0.1	-5.8	1.1±0.1	1.2±0.2	10.8	0.331
IL-18 (ng/L, n = 40)	279.3±15.6	269.3±13.2	-3.6	282.2±14.6	279.0±12.9	-1.1	0.556
MDA (μM, n = 40)	2.3±0.1	2.3±0.1	1.5	2.4±0.1	2.4±0.1	-0.1	0.614
ox-LDL (pg/L, n = 37)	128.6±16.3	127.1±16.8	-1.2	122.0±15.6	122.8±15.7	0.7	0.740

^1^ The results display the mean ± SEM, based on results from both the BC- and the CB subject groups (i.e. both the test group starting with berry beverage and consumed the control beverage in the second intervention period (BC group) and subjects starting with control and consumed the berry beverage in the second intervention period (CB group). Statistical evaluations are performed with analysis of variance (one-way ANOVA, general linear model in Minitab).

^2^ B_0_: baseline values collected at start of the berry intervention.

^3^ B_5wk_: values collected after completion of the berry intervention.

^4^ Changes (%) in test variables between B_5wk_ compared with B_0_.

^5^ C_0_: baseline values collected at start of the control intervention.

^6^ C_5wk_: values collected after completion of the control intervention.

^7^ Changes (%) in test variables between C_5wk_ compared to C_0_.

^8^ *P*-values for the comparison of changes between [B_5wk_—B_0_] and [C_5wk_—C_0_], i.e. differences between changes after berry treatment compared with changes after control treatment.

^9^ *P* = 0.063

*: *P* < 0.05,

**: *P* < 0.01 with respect to differences from baseline concentrations B_0_ and C_0_, respectively, and after 5 wk treatments with berry beverage (B_5wk_) and control beverage C_0_, respectively.

BP: blood pressure. HOMA-IR: homeostatic model assessment of insulin resistance [29].

## Discussion

In this study we investigated effects on cognitive functions and cardiometabolic risk variables following 5 wk intervention with a berry beverage based on a mixture of Swedish berries known to be rich in polyphenols or carotenoids (lycopene). The effects were compared with the effects of a control beverage matched with respect to monosaccharide content and distribution, pH, and volume. The berry beverage resulted in a modest (~5%) but significant improvement in the WM test in comparison with the control beverage at the test point 30 min. In addition, the berry beverage significantly reduced the concentrations of total-C and LDL-C. No significant effects were detected following 5 wk berry intervention on fasting glucose- or insulin concentrations. On the contrary, the control beverage resulted in significantly increased fasting glucose concentrations from baseline. Further, there was a strong tendency towards an increase (~13%) also in fasting insulin concentrations from baseline after the control beverage, which resulted in a significantly increase in fasting insulin concentrations post the control intervention in comparison to the berry intervention (~19% differences in effects between berry- compared with control intervention). An increased insulin resistant after the control treatment compared with the berry treatment was supported by a significantly increased insulin resistant, as determined with HOMA-IR.

Berries are commonly known to contain high levels of a range of phenolic compounds, including a variety of anthocyanins, flavonols, flavanols, proanthocyanidins, ellagitannins, and phenolic acids. The choice of berries included in the berry beverage in the present study was based on literature data indicating high amounts of these compounds [30]. Considerable amounts of polyphenols in the berry beverage was confirmed by the analysis of the test- and control products. Anthocyanins are widely distributed in berries and constitute the strong pigments responsible for the red, blue or purple color of some berries, and additionally act as powerful antioxidants [31]. Anthocyanins were also one of the phenolic groups found in high concentration in the berry beverage (414.2 mg/l). The concentration of anthocyanins was similar to what previously has been observed in blackcurrant juice (435.6–512.7 mg /l) [32], but higher in comparison to what has been found in black grape- or sour cherry juices (92.4–105.5 and 235.1–274.5 mg/l, respectively) [32]. Other groups of phenolic compounds found in considerable concentration in the berry beverage were the flavonols (155.9 mg of flavonols/l).

The antioxidant capacity of the berry beverage as determined by the TEAC method in this study was high (20.3 mmol Trolox/l). For instance, reported TEAC values for other berry beverages such as Cannonau wine, Myrtle liqueur and strawberry-tree honey were lower compared to the currently studied berry mix (9.3, 11.5 and 5.9 mmol Trolox/l, respectively) [33], and lower also in orange juice (5.4 mmol Trolox/l) [34]. Also blueberry, cranberry, goji, açai and pomegranate juices presented lower values of TEAC compared to the berry beverage in this study, being 15.1, 11.5, 7.7, 9.3 and 4.1 mmol Trolox/l, respectively [35]. Furthermore, the FC values of the berry wines were in the same range as for the presently described berry beverage (805.7 mg GAE/l), being for blackcurrant wine from 520 to 1820 mg GAE/l, blackcurrant and strawberry wine from 655 to 950 mg GAE/l, black and red currant wine from 515 to 1270 mg GAE/l, and black and red currant and strawberry wine from 720 mg GAE/l [36].

Metabolic effects of berries, berry extracts, polyphenols and carotenoids, have mostly been evaluated in animal models, e.g. rat and/or mice models. In such studies, the berries included in the presently described study have individually shown benefits on several of the risk markers determined in the present study, e.g. blood pressure, lipid profile, inflammation, and oxidative stress (see e.g. [37–42]). The beneficial effects were to a major part attributed to the polyphenols present. In humans, CVD [43] and T2DM [44] protective effects of berries are supported by prospective studies. Less information is available concerning cardiometabolic effects in controlled berry interventions in humans, especially in healthy people, and results from available studies show conflicting results. However, accumulating evidence indicate improvements of risk factors for T2DM and CVD. For example, a meta-analysis [45] including 22 RCTs (intervention periods between 2–24 wk) investigating effects of different berries (including also the berries studied in the present study) in both healthy and subjects with CVD, showed that berries have the potential to improve cholesterol profile, blood pressure, fasting glucose, BMI, HbA1c, and inflammatory markers. Furthermore, in an acute study setting a strawberry load reduced postprandial insulin concentrations in overweight adults [46], and in normal- or overweight healthy subjects, a load of blackcurrant and lingonberries [47], or a purée with a mixture of berries (bilberries, blackcurrants, cranberries, and strawberries), lowered the postprandial blood glucose response to a sucrose challenge [48]. In the case of tomatoes, except for containing polyphenols, tomatoes contain considerable amounts of carotenoids, especially in the form of lycopene, a bioactive compound with a potent antioxidant capacity. Several studies are in support of lycopene in prevention of CVD, and e.g. reduce levels of ox-LDL, total-C, and blood pressure (reviewed in [49]).

Fasting glucose- and insulin concentrations are less prone to be affected by diet interventions, especially in healthy subjects, and previous studies have shown inconsistent results. Thus, a meta-analysis of 12 RCT (7 interventions in subjects with normal fasting values and 5 in hyperglycaemic subjects) did not show effects of fruit- or berry juices on glucose- and insulin concentrations at fasting in the total group or divided in subgroups of normo- or hyperglycaemic subjects. Neither were there any effects on fasting glucose- or insulin concentrations in subgroups of type of juice (berries, grapes, pomegranate, and orange) [50]. The lack of significant effects is in accordance with the results of the berry beverage on fasting glucose- and insulin concentrations obtained in the presently describe study. However components in the berry beverage matrix seem to have the potential to inhibit an adverse effect of a daily intake of the monosaccharides included in the berry beverage. The daily portions of berry beverage were based on 350 g frozen berries of which blueberry was included in highest amounts (150 g blueberry, 50 g elderberry, 50 g lingonberry, 50 g strawberry, 50 g black currant, and tomatoes (tomato powder, corresponding to 100 g fresh tomatoes)). The control beverage was mainly composed of water and monosaccharides (glucose and fructose). Even though berries in general are considered to be of benefits to health, this amount of berries on a daily basis for 5 wk probably adds to the habitual intake of low molecular weight carbohydrates. The berry- and the control beverages contained equal amounts and type of available carbohydrates (approximately 13g glucose and 20g fructose each day). It can be suggested that the daily amount of monosaccharides in the control beverage may have had an adverse effect on insulin sensitivity and glucose regulation, and, since no such negative effects were observed of the berry beverage, it can be put forward that the berries had the potential to blunt an adverse effects of the daily monosaccharide intake. Further; it can be speculated that the presence of polyphenols (potential effects summarized in ref [51]) in the berry mixture might have been involved in the control of glucose homeostasis, thus counteract negative effects. In this context it should be noted that in addition to polyphenols, berries are a source of other bioactive compounds with potential cardiometabolic benefits. For example, berries are rich in DF, both soluble and insoluble, and accumulating evidence from both epidemiological studies [52] and clinical interventions [53, 54] are at hand proposing beneficial effects of DF on cardiometabolic risk, e.g. with respect to glucose homeostasis. The beneficial effects of DF are to largely suggested to emanate from events related to colonic fermentation [53, 54]. The daily portion of DF from the berry beverage in the current study was approximately 11g.

In respect to cognitive functions, prospective studies indicate that higher intake of blueberries, strawberries [14], and carotenoids (lycopene) [55] is associated with slower rates of cognitive decline in elderly subjects. Beneficial cognitive effects of berries are also supported from intervention studies. Consequently, 12 wk intervention with concord grape juice [56] or blueberry juice [57] demonstrated enhanced cognitive functions (wordlist recall respective wordlist recall and paired associate learning) in elderly subjects with mild cognitive impairment. Lycopene showed to improve cognitive functions in rat models of insulin resistance, in parallel with improvement of insulin signaling deficits, oxidative stress and neuroinflammation [58]. Insulin and insulin receptors within the brain are important for learning and memory. Insulin resistance may occur also in the brain and results in reduced central insulin signaling, altering a variety of insulin mediated events of importance for memory functions [59]. It can be speculated that the improved performance in the WM test after the berry beverage compared to the control beverage, at least partly, may be a consequence of a superior insulin sensitivity and/or insulin receptor signaling in the brain.

After ingestion, polyphenols are present in the circulation mainly as polyphenol metabolites. In human blood brain barrier models [60], both anthocyanins and flavonols, and their metabolites, have been shown to cross the blood brain barrier and can be found in various brain regions important for learning and memory. The mechanisms behind beneficial effects of polyphenols and/or lycopene on cognitive functions and on cardiometabolic risk are not fully elucidated. Further, in the light of the molecular diversity of dietary polyphenols and their metabolites, the possible cellular signaling pathways and mechanisms of action are probably multiple. In addition, substantial parts of the ingested polyphenols are not absorbed into the circulation but instead, like DF, pass to the colon. In the colon, polyphenols are metabolized to other compounds, such as phenolic acids, and the metabolites may be absorbed into the circulation, and may enter the brain. Further, polyphenols and/or their metabolites have been shown to interfere with the gut microbiota composition and metabolism, and may modulate release of bacterial metabolites, such as short chain fatty acids (SCFA) [61]. Consequently, polyphenols entering the gut can be suggested to promote benefits on metabolism and brain functions through promoting a more favorable gut flora and/or release of bacterial metabolites, e.g. SCFA. It has been demonstrated that SCFA in the gut stimulate the release of gut hormones, e.g. GLP-1 [62], an incretin and a neuropeptide with neuroprotective effects, and with beneficial effects on cognitive functions, e.g. learning and memory [63], suggesting possible additional mechanisms regarding cognitive benefits related to colonic fermentation events.

Thus, it can be expected that there are several underlying mechanisms whereby berries potentially may elicit metabolic- and cognitive health effects. However, with respect to polyphenols and lycopene, several effects of importance are proposed to derive from the well-described anti-inflammatory [64] and/or anti-oxidative [65] properties. Low-grade chronic inflammation and oxidative stress has been advocated as important factors behind lifestyle related disorders and diseases, such as obesity, the metabolic syndrome, T2DM, and CVD [66, 67]; risk factors which increasingly are associated with neurodegenerative disorders and cognitive decline [68]. The brain is particularly vulnerable to oxidative stress and inflammation. The susceptibility to oxidative stress and neuro-inflammation further increases with age. With age, there is a decrease in the endogenous antioxidant defense, and at the same time there is an increase in inflammatory mediators. However, despite considerable amounts of polyphenols on a daily basis during 5 wk, markers of inflammation or oxidative stress following the berry beverage in this study did not decrease significantly as judged from analysis of IL-6 (-5.8%) and IL-18 (-3.6%), respective ox-LDL (-1.2%) and MDA (+1.5%).

This study involves some potential study limitations. An evident constraint is the unbalanced gender participation, since 75% of the volunteers were women. Another potential limitation is that the subjects ingested the intervention products at home, not allowing control of compliance. In addition it was not possible to blind the products to the test subjects due to the obvious differences. In this respect it must be noted that it is a common limitation when “real” foods are included in interventions; it is often a challenger (or impossible) to blind the products to the subjects. It is difficult to include “inert” control products without possibly metabolic benefits. Carotenoids were not analyzed in the present study, which can be considered to be an additional potential study limitation. Moreover, the test product included a mix of berries which rule out possibilities to make any conclusions regarding individual berries.

*In conclusion*, 5 wk consumption of a mix of blueberries, strawberries, blackcurrant, elderberries, lingonberries, and tomatoes reduced total-C and LDL-C and prevented an adverse effect of monosaccharides on glucose homeostasis and insulin resistance, in parallel to eliciting an enhanced effect on working memory capacity. The results thus support previous observations regarding health benefits of berries, and propose a preventive potential with respect to the MetS related diseases. The results are also in support of data indicating that diets with benefits on cardiometabolic risk markers in parallel are beneficial to cognitive functions. The beneficial effects of berries are probably related to the bioactive compounds included, such as polyphenols/carotenoids and/or DF. Further studies are needed to clarify the underlying mechanisms.

## Supporting information

S1 Checklist The supporting CONSORT checklist. (DOC)

S1 Text Study protocol (ethical application, the original Swedish version). (DOCX)

S2 Text Study protocol (ethical application, google transalated from Swedish to English). (DOCX)

S3 Text Study protocol to experts in the ethical committee (both in Swedish and google translated into English). (DOCX)
