# Supplementary material for: Effects of a mixed berry beverage on cognitive functions and cardiometabolic risk markers; A randomized cross-over study in healthy older adults
Source: PLoS One. 2017 Nov 15;12(11):e0188173. doi: 10.1371/journal.pone.0188173 (PMC5687726; doi:10.1371/journal.pone.0188173)
Supplement: S1 Text — (DOCX) [file pone.0188173.s002.docx]

# ANSÖKAN OM ETIKPRÖVNING

# Information till ansökan, *se bilaga och Vägledningar (*[*www.epn.se*](http://www.epn.se)*)*

**Till Regionala etikprövningsnämnden i:** Lund

Den regionala etikprövningsnämnd till vars upptagningsområde forskningshuvudmannen hör, se respektive nämnd *(*[*www.epn.se*](http://www.epn.se)*)*

Avgift inbetald datum:

Observera att en ansökan aldrig är komplett och därmed kan behandlas förrän blanketten är korrekt ifylld och avgiften är betald.

**Projekttitel:** Effekter av livsmedel med högt kostfiberinnehåll på metabolism och kognitiva funktioner

Ange en beskrivande titel på svenska för lekmän, utan sekretesskyddad information. Ange också i förekommande fall projektets identitet, projektets/forskningsplanens (protokollets eller prövningsplanens) nummer, version, datum osv.

Projektnummer/identitet:       Version nummer:

## **Uppgifter som fylls i av den regionala etikprövningsnämnden**

Ansökan komplett: Dnr:

Begäran om ytterligare information (i sak): Begärd information inkommen:

Beslutsdatum: Expeditionsdatum:

**Ansökan avser (gäller även vid begäran om rådgivande yttrande):**

Forskning där endast en forskningshuvudman deltar (5 000 kr)

Forskning där mer än en huvudman deltar (16 000 kr)

Forskning där mer än en forskningshuvudman deltar, men där samtliga

forskningspersoner eller forskningsobjekt har ett omedelbart

samband med endast en av forskningshuvudmännen (5 000 kr)

Endast behandling av personuppgifter (5 000 kr)

Forskning som gäller klinisk läkemedelsprövning (16 000 kr)

Ändring av tidigare godkänd ansökan enligt 4 § förordning (2003:615) om

etikprövning av forskning som avser människor (2 000 kr)

Om nämnden finner att forskningsprojektet inte faller inom etikprövninglagens tillämpningsområde

önskas ett rådgivande yttrande. [(Info: 4a och 4b §§ i förordning 2003:615)](http://www.epn.se/media/8604/2003_615_4ab.doc) [(Info: Bilaga till ansökan)](http://www.epn.se/media/8525/bilaga.doc)

Ja:  Nej:

**1. Information om forskningshuvudman m.m.**

**1:1 Forskningshuvudman** ([Info: p. 1:1 i Vägledning till ansökan](http://www.epn.se/media/8601/vta_p1_1.doc))

Ansökan om etikprövning av forskning ska göras av forskningshuvudmannen. *Med forskningshuvudman avses en statlig myndighet eller en fysisk eller juridisk person i vars verksamhet forskningen utförs.* Inom staten utförs forskning främst vid lärosätena, men även vid vissa andra myndigheter, som t.ex. Brottsförebyggande rådet och Socialstyrelsen. Kommuner och landsting kan vara forskningshuvudmän, liksom privaträttsliga juridiska personer.

Namn: Lunds Universitet

Adress: Getingevägen 60, Box 124, 221 00 Lund

**1:2 Behörig företrädare för forskningshuvudmannen**

Behörig företrädare är t.ex. prefekt, enhetschef, verksamhetschef. Forskningshuvudmännen bestämmer själva, genom interna arbets- och delegationsordningar eller genom fullmakt, vem som är behörig att företräda forskningshuvudmannen. Kopia av sådan handling *ska* bifogas.

Namn: Ann-Charlotte Eliasson Tjänstetitel: Prefekt

Adress: Inst. för Livsmedelsteknik, Lunds Universitet, Box 124, 221 00 Lund

**1:3 Forskare som är huvudansvarig för genomförandet av projektet (kontaktperson)**([Info: p. 1:3 i Vägledning till ansökan](http://www.epn.se/media/8714/vta_p1_3.doc))

Namn: Anne Nilsson Tjänstetitel: Teknisk doktor, forskas assistent

Adress: Enheten för Industriell Näringslära och Livsmedelskemi, Inst för Livsmedelsteknik, Lunds Universitet, Box 124, 221 00 Lund

E-postadress: anne.nilsson@appliednutrition.lth.se

Telefon: 046-2228343

Mobiltelefon: 0763 100350

**1:4 Plats** ([Info: p. 1:4 i Vägledning till ansökan](http://www.epn.se/media/8717/vta_p1_4.doc))

Plats (er) där projektet ska genomföras, ange inrättning (ar), institution (er), klinik (er) etc.

Enheten för Industriell Näringslära och Livsmedelskemi, Inst för Livsmedelsteknik, Lunds Universitet, Box 124, 221 00 Lund

**1:5 Andra medverkande**

Övriga deltagande forskningshuvudmän samt forskare ansvariga för att lokalt genomföra projektet (kontaktpersoner) anges här eller i bilaga med namn och adresser (se p. 9 bilaga nr 1).

**1:6 Ansökan/anmälan till andra myndigheter**

**Vid läkemedelsprövning**

Ansökan om tillstånd av *Läkemedelsverket* – se Läkemedelsverkets hemsida ([www.mpa.se](http://www.mpa.se/) )

Ansökan inlämnad (datum)       Tillstånd erhållits

EudraCT nr:

**Vid viss genetisk forskning**

Om personuppgifter om genetiska anlag som har framkommit efter genetisk undersökning kommer att hanteras i studien ska detta anmälas till *Datainspektionen* enligt 10 § personuppgiftsförordningen (1998:1191) – se Datainspektionens hemsida [www.datainspektionen.se](http://www.datainspektionen.se/lagar-och-regler/personuppgiftslagen/forhandskontroll/)

Anmälan inlämnad (datum)       Kommer att inlämnas efter godkänd etikprövning

**Vid viss forskning som innefattar bestrålning av forskningspersoner** ([Info: p. 9 i Vägledning till ansökan](http://www.epn.se/media/8747/vta_p9.doc))

Ansökan, enligt 16 och 22 §§ Strålsäkerhetsmyndighetens föreskrifter (SSMFS 2008:35) om allmänna skyldigheter vid medicinsk och odontologisk verksamhet med joniserande strålning, till *Strålskyddskommitté* –
för vidare information kontakta aktuell lokal strålskyddskommitté.

Ansökan inlämnad (datum):       Ansökan tillstyrkt

### 2. Uppgifter om projektet

**2:1 Sammanfattande beskrivning av forskningsprojektet (programmet)**[Vägledning till forskningsplan/forskningsprotokoll (program)](http://www.epn.se/media/8929/vägledning%20till%20forskningsplan.pdf) ([Info: p. 9 i Vägledning till ansökan](http://www.epn.se/media/8747/vta_p9.doc))

Beskrivningen ska kunna förstås av nämndens samtliga ledamöter. Undvik därför terminologi som kräver specialkunskaper. Ange bakgrund och syfte för studien samt den/de vetenskapliga frågeställning (ar) som man söker svar på. Ange de viktigaste undersökningsvariablerna. Beskriv vilka kunskapsvinster projektet kan förväntas ge och betydelsen av dessa. Ange om det är en registerstudie, uppdragsforskning etc. För fackmän avsedd detaljerad information i forskningsplan/forskningsprotokoll (program) *ska* bifogas som bilaga (se p. 9 bilaga nr 2). En utförligare beskrivning av studiens genomförande *avsedd för lekmän* kan vid behov bifogas den för fackmän avsedda obligatoriska forskningsplanen.

Det metabola syndromet är ett samlingsnamn för ett tillstånd med allvarlig risk för diabetes och hjärt- kärlsjukdomar och inkluderar faktorer såsom förhöjda blodsocker- och insulinnivåer, förhöjda blodfetter, högt blodtryck och bukfetma. En ökad grad av kronisk inflammation och en förhöjd oxidativ stress anses vara starkt knutet till dessa ohälsotillstånd. Det finns numera starka indikationer på att det metabola syndromet och åldersdiabetes även ökar risken för en nedsatt mental (kognitiv) prestationsförmåga. Även ett normalt åldrande leder dock till en nedgång i kognitiv prestationsförmåga.

En kolhydratrik kost som resulterar i en låg och jämn blodglukosstegring (livsmedel med lågt glykemiskt index, GI) har visats ha en positiv effekt vid förebyggande och behandling av åldersdiabetes, hjärt- kärlsjukdomar och det metabola syndromet. En produkt med högt GI ger å andra sidan en snabb och hög höjning av glukoskoncentrationen, vilket har en ohälsosam effekt på dessa tillstånd. Efter intag av ett livsmedel med lågt GI brukar glukoskoncentrationen sjunka långsammare och därefter även komma att ligga på ett högre värde i ett senare skede efter måltiden jämfört med ett hög-GI livsmedel. Detta anses ha gynnsamma effekter på ämnesomsättningen (metabola effekter), men kan även hypotetiskt vara gynnsamt för den kognitiva prestationsförmågan.

Vissa låg-GI livsmedel, t ex hela kornkärnor, har visats ha gynnsamma effekter på blodsockret inte bara akut efter en måltid utan även efter nästkommande måltider. Denna s.k "second-meal effect" har visats både från frukost till lunch (Nilsson et al. 2008), från frukost till middag (Nilsson et al. 2008) och från en sen middag till frukost dagen därpå (Nilsson et al. 2006 och 2008). Förklaringen till den akuta blodsockersänkningen efter en måltid ligger i en förlångsammad nedbrytning av kolhydraterna och absorption av glukos. Detta leder till att det tar längre tid för glukos att komma in i blodbanan varvid blodsockerhöjningen blir lägre och mer utdragen. Förklaringen till "second-meal effekten" från frukost till lunch har även den förklarats av en mer utdragen matspjälkning och absorption vilket ger en förlängd sänkning av fria fettsyror, med en åtföljande höjning av insulinkänsligheten vid intag av lunchen. Orsaken till "second-meal effekten" i ett längre tidsperspektiv, t.ex. från frukost till middag eller från en kvällsmåltid till frukost dagen därpå, är ej helt klarlagd, men troligtvis involverar de bakomliggande mekanismerna bakteriell fermentering av odigererbara kolhydrater i tjocktarmen (kolon). Odigererbara kolhydrater är kolhydrater som ej bryts ner i och tas upp i tunntarmen utan fortsätter till tjocktarmen och utgör där näringssubstrat till bakterier. I tidigare studier har vi visat att intag av en kornbaserad måltid positivt kan påverka glukostolerans och andra riskmarkörer för metabolt syndrom, så som markörer för inflammation (IL-6, adiponektin) och mättnad i ett 10-12 h perspektiv (Nilsson et al. 2006 och 2008). Denna förbättring var korrelerad till en ökad kolonfermentering mätt med vätgas i utandningsluften samt en ökad produktion av kortkedjiga fettsyror i kolon (SCFA mätt i plasma). Vidare såg vi på morgonen efter en kornbaserad kvällsmåltid en ökad koncentration i plasma av inkretin-hormonet GLP-1 (ett hormon som frisätts i tarmen) ; GLP-1 korrelerade negativt till blodglukosresponsen efter frukostmåltiden. GLP-1 utövar ett flertal metabola funktioner som ger effekter på glukosregleringen. Förutom effekter på glukosregleringen reducerar även GLP-1 magsäckstömningshastigheten vilket kan öka mättnadskänslan och reducera energiintaget. Pga de positiva effekterna på glukosreglering och mättnad har GLP-1 på senare år därför beskrivits som ett "anti-diabetiskt" hormon. Förslag finns att odigererbara kolhydrater antingen direkt eller indirekt via fermentering kan stimulera frisättningen av GLP-1. Vilka bakterier som i så fall utövar den positiva effekten vet man inte. Kolonfermenteringen (vätgashalten i utandningsluften) i vår tidigare studie korrelerade positivt till mättnad och negativt till magsäckstömningshastigheten.

Vår hypotes är att odigererbara kolhydrater kan ha en hälsosam effekt på metabolismen och i förebyggandet av metabolt syndrom och diabetes typ 2 genom mekanismer som härstammar från fermentering av kolonbakterier av odigererbara substrat, och att denna effekt skiljer sig beroende av val av substrat.

Det är välbekant att diabetes och metabolt syndrom leder till en ökad risk för nedsatt kognitiv prestationsförmåga. Vi har i tidigare studier visat att graden av glukostolerans kan påverka kognitiv prestationsförmåga även inom gruppen av personer med normal glukostolerans (Nilsson et al. 2009). Hjärnan är beroende av insulin och god insulinreceptorsignalering för att fungera optimalt. Vid insulinresistens, så som uppkommer vid metabolt syndrom och diabetes, minskar insulinkoncentrationen och insulinreceptorsignaleringen i hjärnan och påverkar därför hjärnans kognitiva kapacitet negativt. En förbättrad insulinkänslighet och glukostolerans, som har visats t ex efter intag av kornbaserade produkter, kan därför tänkas ha positiva effekter även på kognitiv prestationsförmåga.

Det aktuella projektet är en del i ett forskningsprogram (Antidiabetic Food Center, AFC). Den övergripande målsättningen i detta forskningsprojekt är att öka kunskapen för att möjliggöra design av livsmedel som har en positiv inverkan på riskfaktorer relaterade till fetma, åldersdiabetes, och hjärt-kärl- sjukdomar. Syftet med projektet för vilken den aktuella ansökan avser är att studera sambandet mellan bakteriefermentering i kolon av odigererbara substrat och systemisk metabolism samt kognitiv prestationsförmågan.

Kolonsubstrat som ska studeras är:

•Produkter som naturligt innehåller hög halt av odigererbara kolhydrater, så som cerealier, legymer, frukt och bär.

•Produkter (t ex vitt bröd) som berikats med naturliga odigererbara substrat så som kostfiber, resistent stärkelse och polyfenoler, som isolerats från källor innehållande dessa fermenterbara substrat så som cerealier, legymer, frukt och bär.

Projektet genomförs som delstudier på avdelningen för Industriell Näringslära och Livsmedelskemi, Lunds Universitet.

a) kartläggning av metabola och/eller kognitiva effekter av testprodukter då produkten intas på kvällen och testparametrar mäts vid en efterföljande standardiserad frukost.

b) kartläggning av metabola och/eller kognitiva effekten vid en standardiserad frukost då en testprodukt intagits under flera dagar (upp till 1 vecka).

c) på samma sätt som a och b men probiotiska bakterier tillsätts testmåltiderna (t ex lactobaciller och bifidobakterier (bakterier som tillsätts t ex ProViva och mejeriprodukter).

Dvs, sammantaget blir det fyra delstudier (a, b, ca, cb). Varje delstudie innehåller totalt upp till 4 serier. Antalet testprodukter i en serie är mellan 1-5 st. Dessutom ingår en referensprodukt (vitt bröd utan tillsatt fiber) i varje delstudie. I varje delförsök ingår mellan 17 försökspersoner (då inga kognitiva parametrar mäts) och 40 försökspersoner (då kognitiva tester ingår). En "cross-over" design används, dvs varje försöksperson testas efter samtliga testprodukter och referensprodukt (en produkt i taget), och varje testpersons resultat efter samtliga produkter jämförs inom samma testperson. Testprodukterna intas i en randomiserad ordning med ca 1 v mellan två produkter för att säkerställa att inte det föreligger någon kvardröjd effekt av en tidigare produkt.

Sammanlagt i en delstudie deltar varje testperson i försök vid högst vid 6 tillfälle (fem produkter + en referensprodukt). På försöksdagarnas morgon 07.45 kommer testpersonerna fastande från kvällen innan (21.00) då en testprodukt eller referensprodukt intagits. Fastande och upprepat under tre timmar efter den standardiserade frukosten testas försökspersonerna (blodprov, vätgas i utandningsluft (markör för kolonfermentering), registrering av subjektiv mättnadskänsla, kognitiv prestationsförmåga). Faeses prover lämnas före och efter intag av respektive testprodukt för kartläggning av bakterieflora i kolon.

**2:2 Vilken/vilka vetenskaplig (a) frågeställning (ar) ligger till grund för projektets utformning?**

Om projektet kan karakteriseras som en hypotesprövning, ange den primära och eventuellt sekundära hypotesen. Hänvisning till mer detaljerad information för fackmän kan ske till bifogad forskningsplan enligt punkt 2:1

Det aktuella projektet är en del i ett forskningsprogram (Antidiabetic Food Center, AFC). Den övergripande målsättningen i detta forskningsprojekt är att öka kunskapen för att möjliggöra design av livsmedel som har en positiv inverkan på riskfaktorer relaterade till fetma, åldersdiabetes, och hjärt-kärl sjukdommar. Allt mer data tyder på att det föreligger ett samspel mellan microbiota i tarmen och utveckling av låggradig kronisk inflammation, fetma och andra metabola störningar (Cani et al. 2009). Vår hypotes för projektet vilken den aktuella ansökan avser är att odigererbara kolhydrater kan ha en hälsosam effekt på metabolismen och i förebyggandet av metabolt syndrom och diabetes typ 2 genom mekanismer som härstammar från fermentering av kolonbakterier av odigererbara substrat, och att denna effekt skiljer sig beroende av val av substrat. Syftet med projektet för vilken den aktuella ansökan avser är att studera sambandet mellan bakteriefermentering i kolon av odigererbara substrat och systemisk metabolism samt kognitiv prestationsförmågan.

**2:3 Redogör för resultat från relevanta djurförsök**

Om djurförsök inte utförts ange skälen till detta.

Djurförsök är inte relevant för dessa typer av studier.

**2:4 Redogör översiktligt för undersökningsprocedur, datainsamling och datas karaktär**

([Info: p. 2:4 i Vägledning till ansökan](http://www.epn.se/media/8720/vta_p2_4.doc))

Av beskrivningen ska framgå hur projektet planeras genomföras. Beskriv insamlade datas karaktär. Ange hur datas tillförlitlighet säkerställs (t.ex. kvalitetskontroll/monitorering). - Vid enkäter och intervjuer ska beskrivas tillvägagångssätt och t.ex. frågors innehåll och hur slutsatser dras. Enkäter och skattningsskalor *ska* bifogas (se p. 9 bilaga nr 5). - För medicinsk forskning ska anges t.ex. typer av ingrepp, mätmetoder, antal besök, tidsåtgång vid varje försök, doser och administrationssätt för eventuella läkemedel och/eller isotoper, blodprovsmängd (även ackumulerad mängd vid multipla försök). Ange även om och på vilket sätt undersökningsprocedur m.m. skiljer sig från klinisk rutin. Ange proceduren för att ge den eventuella behandling efter projektets slut, som kan erfordras. Ange procedur för insamling av biologiskt material. Redogör för datakällor och procedurer vid behandling av personuppgifter. För mer detaljerad information kan hänvisning ske till bilagd forskningsplan.

Projektet ska genomföras som delstudier på avdelningen för Industriell Näringslära och Livsmedelskemi, Lunds Universitet:

a) kartläggning av metabola och/eller kognitiva effekter av testprodukter då produkten intas på kvällen och testparametrar mäts vid en efterföljande standardiserad frukost.

b) kartläggning av metabola och/eller kognitiva effekten vid en standardiserad frukost då en testprodukt intagits under flera dagar (upp till 1 vecka).

c) på samma sätt som a och b men probiotiska bakterier tillsätts testmåltiderna (t ex lactobaciller och bifidobakterier (bakterier som tillsätts t ex ProViva och mejeriprodukter).

Antalet testprodukter i en delstudie är mellan 1-5 st. Dessutom ingår en referensprodukt (vitt bröd utan tillsatt fiber) i varje delstudie. I varje delförsök ingår mellan 17 försökspersoner (då inga kognitiva parametrar mäts) och 40 försökspersoner (då kognitiva tester ingår). En "cross-over" design används, dvs varje försöksperson testas efter samtliga testprodukter och referensprodukt (en produkt i taget), och varje testpersons resultat efter samtliga produkter jämförs inom samma testperson. Testprodukterna intas i en randomiserad ordning med ca 1 v mellan två produkter för att säkerställa att inte det föreligger någon kvardröjd effekt av en tidigare produkt.

Sammanlagt i en delstudie deltar varje testperson i försök vid högst vid 6 tillfälle (fem produkter + en referensprodukt). På försöksdagarnas morgon 07.45 kommer testpersonerna fastande från kvällen innan (21.00) då en testprodukt eller referensprodukt intagits. Fastande och upprepat under tre timmar efter den standardiserade frukosten testas försökspersonerna.

Blodprover

Kapillärt blodprov tas för blodglukosbestämning fastande och sedan upprepat (upp till åtta gånger) under en tretimmars period. Postprandiella blodglukossvängningar, så som efter en måltid, analyseras med fördel på kapillärt blod, ej på venöst. En venflon sätts i en ven i armvecket och venösa prover tas genom denna vid samma tidpunkter som för blodglukos. Blodprover analyseras med avseende på: blodglukos, insulin, tarmhormoner (GIP, GLP-1), FFA, SCFA, antioxidativ kapacitet (så som SOD, katalas, glutationperoxidas), inflammations markörer (så som IL-6, CRP, adiponektin) och mättnadsmarkörer (så som Grelin, CCK, PYY, PP). Den sammanlagda mängden blod per försöksdag blir < 80 ml. Den sammanlagda mängden blod under ett försök blir högst 500 ml (samlat under 1,5-2 månader).

Faecesprover

Faecesprover kommer få lämnas för att kartlägga kolonflora.

Prover på utandningsluft

Prover på vätgas i utandningsluften (markör för kolonfermentering) tas vid samma tidpunkter som blodprovstagningen genom att försökspersonerna får andas ut ett djupt andetag i en portabel vätgasmonitor. Vätgasutsöndringen är ett mått på fermentering i tjocktarmen.

Mättnadskänsla

Mättnadsformulär som beskriver upplevd subjektiv mättnad, hunger och viljan att äta får fyllas i upprepat under 3 h perioden.

Kognitiva tester

De kognitiva testerna som kommer att ingå i studien omfattar testning av arbetsminne (Working memory, WM), uppmärksamhet/koncentrationsförmåga (selective attention (SA-test)), minnestest för korttidsminne, samt stroop test. Person med stor erfarenhet av liknande tester ansvarar för de kognitiva testerna (Fil.dr Karl Radeborg från institutionen för psykologi, LU). WM-testet och SA-testet är liknande de som beskrivs i en tidigare studie utförd i forskargruppen (Nilsson et al. 2009).

WM-test. WM är en kognitiv kapacitet med en begränsad ”arbetsvolym” som samtidigt både kan tillfälligt lagra (ett fåtal ord eller siffror och under en kort tidsperiod) och processa information. WM testet mäter således kapaciteten att samtidigt kunna lagra och processa information. Det finns flera anledningen till att välja WM som ett mått på kognitiv förmåga i den aktuella studien. WM är involverad i flera vardagliga aktiviteter som t ex matematisk problemlösning där man ska hålla i minnet en del-lösning samtidigt som man utför vidare kalkyler. Mätningar av WM har visats korrelera till många skilda aktiviteter såsom läsförståelse, protokollskrivning, följa vägbeskrivningar, resonerande, och komplicerad inlärning. Vissa författare (Kyllonen 1996; Engle, Kane et al. 1999) anser t o m att WM och intelligens (mätt med Ravens Matris) representerar så gott som samma begrepp. Medan intelligenstest ej kan användas mer än en gång pga risk för betydande inlärnings effekter anses det emellertid att kognitiva test av WM kan utföras vid upprepade mätningar.

SA-test: Datoriserat test bestående av 96 bilder av en kvadrat uppdelad på fyra mindre kvadrater. En av de mindre kvadraterna är röd och en är grön och två är ofärgade. Bilderna visas en och en i 2 s på dataskärmen. Försökspersonerna ska komma ihåg var den röda respektive den gröna kvadraten är placerade inom den större kvadraten. När en ny bild dyker upp på skärmen ska försökspersonen så snabbt som möjligt markera, genom att trycka på en av tre möjliga tangenter, om den röda, den gröna, eller ingen av de färgade kvadraterna är kvar på samma plats som i den föregående bilden.

Ordlistor: Minnestest för korttidsminne (episodiskt minne). Ordlistor bestående av 30 ord läses upp med två sek mellanrum. Omedelbart efter att orden läses upp får försökspersonerna 2 min på sig att skriva ner så många av orden de kommer ihåg. Även test av en fördröjd nerskrivning av orden (10-20 min) kan förekomma. Testet bygger på ett välkänt test ”The Rey Auditory-Verbal Learning test” (Vaisman et al. 1996).

Stroop-test: Stroop-testet är utvecklat för att mäta uppmärksamhet och förmågan att fokusera på det som är väsentligt under en längre tid (liknande SA-testet). Testet utförs på dator, och går ut på att en interferens uppstår i hjärnan som beror på att man sätter igång två olika kognitiva processer samtidigt. Ordet på färger är skrivna med text av en annan färg än vad som texten anger. Försökspersonerna ska registrera färgen på texten och ignorera textens betydelse (vilken färg som står skrivet). Interferens uppstår då p.g.a. att en automatisk kognitiv process gör att man läser ett ord man fokuserar på samtidigt som den kontrollerade processen försöker avgöra vilken färg ordet har.

Innan försöken startar ska försökspersonerna få genomföra testerna så de är bekanta med testerna redan innan det första egentliga försökstillfället.

**2:5 Redogör för om insamlat biologiskt material kommer att förvaras i en biobank** ([Info: p. 2:5 i Vägledning till ansökan](http://www.epn.se/media/8723/vta_p2_5.doc))

*Med biobank avses biologiskt material från en eller flera människor som samlas och bevaras tills vidare eller för en bestämd tid och vars ursprung kan härledas till den eller de människor från vilka materialet härrör.*Redogör för var och hur prover som ska sparas förvaras, kodningsprocedurer och villkor för utlämnande av prover. Ange huvudman för biobanken. Observera att i förekommande fall ska anmälan av biobank ske till Socialstyrelsen enligt lagen (2002:297) om biobanker i hälso- och sjukvården m.m.

Insamlat biologiskt material sparas ej i en biobank. När studien är genomförd och blodproverna analyserade avidentifieras proverna. Proverna destrueras när ett manuskript blivit accepterat för vetenskaplig publikation.

**2:6 Redovisa tillgång till nödvändiga resurser under projektets genomförande**

Ange vem/vilka som har ansvaret (prefekt, verksamhetschef eller motsvarande) för forskningspersonernas säkerhet vid alla enheter/kliniker där forskningspersoner ska delta. Intyg från dessa ansvariga *ska* bifogas (se p. 9 bilaga nr 9). Av intyget ska framgå att erforderliga ekonomiska, strukturella och personella resurser finns tillgängliga för att garantera forskningspersonernas säkerhet.

Se bifogat intyg (bil 9).

Blodprover tas av legitimerad sjuksköterska och erfaren laboratoriepersonal. Person med stor erfarenhet av studier med kognitiva tester (docent Karl Radeborg från institutionen för psykologi, LU) ansvarar för testerna.

**2:7 Journalföring, registrering och hantering av data** ([Info: p. 2:7 i Vägledning till ansökan](http://www.epn.se/media/8750/vta_p2_7.doc))

Redogör för hur undersökningsprocedurer och eventuella ingrepp journalförs. Ange hur registrering och behandling av resultaten ska gå till. Om materialet ska kodas, ange proceduren, vem som förvarar kodlistor och vem eller vilka som har tillgång till dem, var och hur länge de förvaras samt om materialet kommer att anonymiseras eller förstöras. Ange om band- och videoinspelningar används. Redogör för vilken tillgänglighet datamaterialet har och hur det förvaras samt hur erforderligt sekretesskydd erhålls.

Vid provtagning kodas försökspersonernas identitet med initialer och proverna kodas med löpnummer (ett nummer ges vid varje tidpunkt för provtagning). Det samma gäller för faecesprover. Resultaten för de kognitiva testen kodas med personernas två första bokstäver för för och efternamn, samt datum för utförandet. Kodlistor förvaras i en pärm på ansvarig forskares kontor (låst). Endast forskaren och provtagaren har tillgång till kodlistorna. Efter att resultaten är utvärderade kommer kodlistorna att avidentifieras.

**2:8 Redogör för tidigare erfarenheter (egna och/eller andras) av den använda
proceduren, tekniken eller behandlingen**

Särskilt angeläget är att redovisning av risker för komplikationer görs tydliga och i förekommande fall med angivande av relevanta publikationer. Vid nya behandlingar av patienter, t.ex. med läkemedel, bör anges hur många patienter (med aktuell eller annan åkomma) som tidigare erhållit föreslagen behandling, läkemedelsdosering (eller annan dosering) samt hur långa behandlingsperioder som studerats.

Riskerna är i stort sett obefintliga. Vid avdelningen för Industriell Näringslära och Livsmedelskemi finns en mycket stor erfarenhet av liknande studier. Många måltidsstudier med kapillära och venösa blodprovstagningar har genomförts av bl a den för det aktuella projektet ansvariga forskaren (ex diarienr: Etik: H4 19/2006 "Effekt på glukostoleransen vid frukost av olika spannmålsprodukter intagna på kvällen"). På samma sätt finns det även erfarenhet av studier på arbetsminne och uppmärksamhet i förhållande till glukosrespons (diarienr: 679/2004).

**3. Uppgifter om forskningspersoner**

**3:1 Hur görs urvalet av forskningspersoner?** ([Info: p. 3:1 i Vägledning till ansökan](http://www.epn.se/media/8726/vta_p3_1.doc))

*Med forskningsperson avses en levande människa som forskningen avser.*Ange urvalskriterier (inklusion och exklusion). Redogör för på vilket sätt forskaren kommer i kontakt med/får kännedom om lämpliga forskningspersoner. Ange om rekrytering sker från egna/andras tidigare eller pågående studier. Om annonsering sker, *ska* annonsmaterialet insändas som bilaga (se p. 9 bilaga nr 3). Om t.ex. barn eller personer som tillfälligt eller permanent inte är kapabla att ge ett eget informerat samtycke ska ingå i projektet, ska detta särskilt motiveras. Om vissa grupper (t.ex. kvinnor, barn eller äldre) utesluts från deltagande i projektet ska detta särskilt motiveras.

Friska personer mellan 20 och 70 år, både män och kvinnor, med BMI<30, kommer att ingå. Kognitiva tester kommer inte att ingå i varje delstudie. När inga kognitiva tester ingår kommer försökspersonerna som rekryteras vara mellan 20-35 år, och rekryteras då genom annonsering på universitetsområdet (Lunds Universitet) (se bilaga 3a) och genom kontakt via mejl eller telefon av forskningspersoner som tidigare deltagit i liknande studier och som meddelat att de vill delta i fler studier. Då kognitiva testningar utförs kommer försökspersonerna vara mellan 50-70 år och rekryteras genom annonsering i dagspressen (se bilaga 3b). Anledningen till att en äldre population rekryteras då kognitiva mätningar ska utföras ligger i frågeställningen för projektet. Risken för metabola störningar ökar med åldern, liksom för risken för nedsatt kognitiv prestationsförmåga. De yngre försökspersonerna är svårare att studera med avseende dels på deras aktuella studievana (universitetsstuderande) och för att de fortfarande är så unga att deras kognitiva fårmåga troligtvis inte har blivit påverkad av ålder och metabolismen.

Ingen allmän hälsoundersökning begärs av försökspersonerna för att kunna delta i studierna. I informationen till försökspersonerna informerar vi om att man som deltagare i studien inte ska ha några kända sjukdomar som t ex stör ämnesomsättningen. Riktigheten i att forskningspersonerna är friska bygger därför till stor del på att forskningspersonerna har givit korrekta uppgifter. De kan naturligtvis ha någon odiagnostiserad sjukdom som de ej vet om själva. Om det visar sig att någon testvariabel (framför allt blodsocker) är utanför gränsen som anses normal kan forskningspersonen ej delta i studien, och resultaten av analyserade prover används ej vid de statistiska beräkningarna. En sjuksköterska informerar forskningspersonen och ger vägledning och lämpliga råd.

**3:2 Ange relationen mellan forskare/försöksledare och forskningspersonerna**

Behandlare (t.ex. läkare, psykolog, sjukgymnast) - forskningsperson (t.ex. patient, klient)

Kursgivare (lärare) - student

Arbetsgivare - anställd

Annan relation som kan tänkas medföra risk för påverkan. Beskriv: Ingen relation finns mellan forskare och forskningspersoner

3:3 Redogör för det statistiska underlaget för studiepopulationens (-ernas)/ undersökningsmaterialets (-ens) storlek [(Info: p. 3:3 i Vägledning till ansökan)](http://www.epn.se/media/8729/vta_p3_3.doc)

Redovisa statistisk styrka, så kallad ”power”- beräkning eller redovisa motsvarande överväganden som tydliggör studiens möjligheter att besvara frågeställningarna.

I en tidigare studie i samma område där mätning av kognitiv prestationsförmåga ingick (diarienr: 679/2004, "Effekter av frukostar med olika postprandialt glukossvar på arbetsminne samt uppmärksamhet") beräknades att 33 försökspersoner skulle vara tillräckligt. Effektstorleken (Cohen´s d) uppskattades då till d=0.50 (vilket Cohen kallar medelstor effekt), vid tvåsidig hypotesprövning en power av 0.77 och vid ensidig hypotesprövning en power av 0.86. En power av 80% brukar inom psykologisk forskning anses tillräcklig för att motivera en undersökning. (Beräkningen av power baseras på tabell 9-9 och 9-10 i Aron & Aron 82003: Statistics for Psychology.) För att kunna designa matchade grupper (test produkt, kvinna/man) ingick 40 försökspersoner i studien, vilket var ett antal som visades vara ett tillräckligt antal. I studier där effekter på kognitiva funktioner ingår ska därför 40 försökspersoner ingå.

Av tidigare liknande studier vi utfört där vi studerat effekter av cerealiebaserade produkter (t ex kornkärnor) på metabola parametrar och testmarkörer för metabolt syndrom i ett övernatten perspektiv och där ej mätning av kognitiva funktioner har ingått har vi med framgång använt mellan 15-20 försökspersoner (t ex Nilsson et al 2006 och 2008). Antalet försökspersoner kommer därför att vara mellan 15-40.

**3:4 Ange om forskningspersonerna kan komma att inkluderas i flera studier samtidigt
eller i annan/andra studie (-er) i nära anslutning till denna? I så fall vilken typ av forskning?** ([Info: p. 3:4 i Vägledning till ansökan](http://www.epn.se/media/8732/vta_p3_4.doc))

nej

3:5 Vilket försäkringsskydd finns för de forskningspersoner som deltar i projektet?

Det åligger forskningshuvudmannen att kontrollera att befintliga försäkringar täcker eventuella skador som kan uppkomma.

Försäkringsskydd finns i form av ett särskilt personskydd som tecknats hos Kammarkollegiet.

**3:6 Vilken ekonomisk ersättning eller andra förmåner utgår till de forskningspersoner
som deltar i projektet och när betalas ersättningen ut?** Utförligare beskrivning kan lämnas i bilaga. ([Info: p. 3:6 i Vägledning till ansökan](http://www.epn.se/media/8735/vta_p3_6.doc))

Ersättning för obehag och besvär. Belopp (före skatt): 300-500 kr per testprodukt

Ersättning för förlorad arbetsinkomst  Ja  Nej

Reseersättning  Ja  Nej

Befrielse från kostnader för läkemedel  Ja  Nej

Befrielse från andra kostnader. Vilka?

Andra förmåner. Vilka? nej

När betalas ersättningen ut? efter avslutad försöksserie. Om försökspersonen avbryter sitt deltagande i förtid får denna ersättningsbelopp som är i proportion till deltagandet.

Ingen ersättning betalas ut

### 4. Information och samtycke ([Info: Forskningspersonsinformation](http://www.epn.se/media/8598/forskningspersonsinformation.doc))

4:1 Proceduren för och innehållet i den *information* som lämnas då forskningspersoner tillfrågas om deltagande

Beskriv hur och när information ges och vad den innehåller. Ange vem som informerar. Normalt ska en kortfattad och lättförståelig skriftlig information ges. Denna skriftliga information *ska* bifogas ansökan (se p. 9 bilaga nr 4). Om ingen eller ofullständig information ges, måste skälen för detta noggrant anges.

Vid första kontakten (projektledare-försöksperson) som brukar ges då försökspersonerna svarar på anonsen ges en kortfattad information angående studien och allmänn orientering görs gällande om försökspersonen är lämplig som försöksperson (frisk och normalviktig och har rätt ålder för att kunna delta). Efter den första kontakten skickas en mer detaljerad information per mejl eller brev (se bilaga 4) angående studien som försökspersonen kan läsa i lugn och ro. Vid den första kontakten ber vi försökspersonen höra av sig igen om hon eller han är fortsatt intresserad av att delta efter att ha läst igenom informationen. Denna kontakt sker via mejl eller telefon. Försökspersonerna får ställa frågor och ev bestämns en tid för startdadum av försöket. Försökspersonerna uppmuntras att kontakta projektledaren, och meddelas att han alltid är välkommen att ta kontakt om det är något som är fortsatt oklart.

**4:2 Hur och från vem inhämtas *samtycke*?**

Beskriv proceduren; vem som frågar, när detta sker och hur samtycket dokumenteras. Utförlig redovisning är särskilt viktig då barn eller personer med nedsatt beslutskompetens ingår i studien, likaså vid studier av en grupp/grupper, t.ex. föreningar, organisationer, företag, kyrkosamfund, församlingar eller skolklasser.

Forskaren som håller i studien skickar via mejl eller brev ut information angående studien till personer som hört av sig efter annonseringen (se bil4 ). Om personen bestämmer sig för att delta undertecknas detta informationsblad vid första träffen mellan försöksperson och försöksledare/projektledare. Samtycke undertecknas i två exemplar varvid forskningepersonen får det ena och forskningsledaren det andra.

### 5. Forskningsetiska överväganden

5:1 Redogör för de risker som deltagandet kan medföra samt möjliga komplikationer

Dessa kan vara t.ex. fysisk skada, smärta, obehag eller integritetsintrång som projektet innebär eller kan innebära. Ange vilka åtgärder som har vidtagits för att förebygga de risker som nämns ovan samt vilken beredskap som finns för att hantera sådana komplikationer. Ange vilka/de metoder som kommer att användas för att efterforska, registrera och rapportera oönskade händelser.

Om det visar sig att någon testvariabel (framförallt blodsocker) är utanför gränsen som anses normalt informerar en sjuksköterska forskningspersonen om detta och ger vägledning och lämpliga råd. Riskerna för komplikationer vid försöken är mycket små. Provsvaren behandlas konfidentiellt. Alla resultat baseras och redovisas på gruppmedeltal. Ingen enskild person kan därför identifieras vid resultatredovisning. Forskningspersonerna är friska, deltar frivilligt och är noga informerade om att de närsom helst kan avbryta försöken utan att ange några skäl.

5:2 Redogör för förutsebar nytta för de forskningspersoner som ingår i projektet

Den nytta som forskningspersonerna kan tänkas ha av studien är baserat på de resultat som studien kan ge. Studien ger ingen annan speciell nytta för de enskilda försökspersonerna.

5:3 Gör en egen värdering av förhållandet risk - nytta för de forskningspersoner som deltar

Försökspersoner uppger ofta att de deltar för att de är intresserade av att bidra till forskning angående kostrelaterad hälsa. En symbolisk summa ges som ersättning vilket för de flesta människor är för lite för att känna sig tvingad att delta på ekonomiska grunder. Riskerna att delta är ringa. Forskningspersonerna bidrar till att viktig kunskap byggs upp. På sikt kan det leda till design av livsmedel med hälsobefrämjande egenskaper.

**5:4 Identifiera och precisera om etiska problem t.ex risk - nytta i ett vidare perspektiv
kan uppstå inom eller genom projektet**

Här kan redovisas om exempelvis vissa grupper kan komma att utpekas/få hjälp som ett resultat av studien.

Det är inte troligt att det kan uppstå etiska problem ur projektet. Nyttan med studien ligger i att resultaten kan föra forskningen framåt i karläggningen av samband mellan diet och hälsa, vilket eventuellt i en förlängning kan påverka folkhälsan. Idag är en stor del av befolkningen överviktiga och lider av metabola störningar, t ex diabetes typ 2. Det innebär ett stort lidande för den enskilda men även en stor kostnad för samhället. Att kunna förstå och sedan förebygga/minska denna ohälsa genom kosten vore därför en stor vinst både för den enskilda och för samhället.

### 6. Redovisning av resultaten

**6:1 Hur garanteras forskningshuvudmannen och medverkande forskare tillgång till data (anges vid t.ex. uppdragsforskning) och vem ansvarar för databearbetning och rapportskrivning?**([Info: p. 6:1 i Vägledning till ansökan](http://www.epn.se/media/8744/vta_p6_1.doc))

Ansvarig för raportskrivning och databearbetning är tekn. dr Anne Nilsson och prof. Inger Björck (Industriell Näringslära och Livsmedelskemi, Lunds Universitet). Ansvarig för databearbetning då det gäller de kognitiva resultaten är även docent Karl Radeborg (inst för psykologi, Lunds Universitet).

6:2 Hur kommer resultaten att göras offentligt tillgängliga? Kommer studien att insändas för publicering i tidskrift eller publiceras på annat sätt?

Ange i vilken form resultaten planeras offentliggöras samt tidsplan för detta.

Resultaten kommer att insändas för publicering i en vetenskaplig tidskrift.

6:3 På vilket sätt garanteras forskningspersonernas rätt till integritet när materialet offentliggörs/publiceras?

Redovisas resultat på statistisk gruppnivå? Beskriv procedurer eller metoder för avidentifiering/anonymisering.

Resultatet redovisas endast på statistisk gruppnivå.

7. Redovisning av ekonomiska förhållanden och beroendeförhållanden

Redovisning enligt punkterna 7:1-7:3 syftar till att tydliggöra alla direkta eller indirekta förhållanden, som kan tänkas påverka forskarens relation till forskningspersonerna (vid t.ex. informations-, samtyckes-, genomförandeprocedurer).

7:1 Vid uppdragsforskning

Ange uppdragsgivaren t.ex. ett företag (vid klinisk läkemedelsprövning eller prövning av andra nya produkter), en organisation eller en myndighet.

Namn:       Kontaktperson:

Adress:       Telefon/mobiltelefon:

Ange uppdragsgivarens relation till forskningshuvudmannen/medverkande forskare, t.ex.
anställningsförhållande

**7:2 Redovisa eventuella ekonomiska överenskommelser med uppdragsgivare eller**

**andra finansiärer (namn, belopp)**

Vid klinisk läkemedelsprövning bör hänvisning ske till ingånget avtal med sjukvårdshuvudmannen. Liknande överenskommelser kan förekomma vid annan uppdragsforskning och ska redovisas på samma sätt. Separata överenskommelser med den/de som ska genomföra forskningen ska redovisas. Belopp som kommer att erhållas för studien/ersättning till kliniken/genomföraren, vad ersättningen ska täcka och ev. belopp som erhålls per forskningsperson, ska också anges här (se p. 9 bilaga nr 12).

7:3 Redovisa forskningshuvudmannens, huvudansvarig forskares och medverkande forskares egna intressen

Här redovisas t.ex. aktieinnehav, anställning, konsultuppdrag i finansierande företag, eget företag som kan få (direkt eller indirekt) ekonomisk vinst av forskningen (se p. 9 bilaga nr 12).

**8. Undertecknande**

Behörig företrädare för sökande forskningshuvudman enligt p. 1:2

Ort: Lund Datum:

Signatur: __________________________________________________________________

Namnförtydligande: Ann-Charlotte Eliasson

Tjänstetitel: Prefekt

Undertecknad forskare som genomför projektet (kontaktperson) enligt p. 1:3 intygar härmed att forskningen kommer att genomföras i enlighet med ansökan

Ort: Lund Datum:

Signatur: _________________________________________________________________

Namnförtydligande: Anne Nilsson

Tjänstetitel: Tek.dr, forskarassistent

**9. Förteckning över bilagor** ([Info: p. 9 i Vägledning till ansökan](http://www.epn.se/media/8747/vta_p9.doc))

Dokument som, i tillämpliga fall, ska bifogas *om inte motsvarande information finns i blanketten* har markerats med x. Markera de bilagor som skickas in med denna ansökan.

| **Insänd med ansökan** | **Bil nr** | Beskrivning | **Klinisk läkemedels-**  **prövning** | **Annan forskning** |
| --- | --- | --- | --- | --- |
|  | 1 | Deltagande forskningshuvudmän och medverkande forskare (kontaktpersoner) vid forskning där mer än en forskningshuvudman deltar. Info p. 1:5 | x | x |
|  | 2 | För fackmän avsedd forskningsplan, vid behov även för lekmän avsedd bilaga. Info p. 2:1 och i Vägledning till forskningsplan/forskningsprotokoll (program) | x | x |
|  | 3 | Annonsmaterial för rekrytering av forskningspersoner. Info p. 3:1 och i Vägledning till ansökan p. 3:1 | x | x |
|  | 4 | Skriftlig information till dem som tillfrågas. Info p. 4:1 och i Forskningspersonsinformation | x | x |
|  | 5 | Enkät, frågeformulär. Info p. 2:4 | x | x |
|  | 6 | Gemensam EU blankett (gäller fr.o.m. den 1 maj 2004), gäller även vid ändring. | x |  |
|  | 7 | Sammanfattning av protokollet på svenska | x |  |
|  | 8 | Prövarhandbok alt. bipacksedel/produktresumé/IB | x |  |
|  | 9 | Intyg från verksamhetschef/motsv. om resurser och om forskningspersonernas säkerhet. Info p. 2:6 | x | x |
|  | 10 | CV för forskare (samma som p. 1:3) med huvudansvar för genomförandet, redovisa forskarens (-arnas) kompetens av relevans för studien. Info i Vägledning till ansökan p. 1:3 | x | x |
|  | 11 | Beskrivning av ersättning till forskningspersoner. Info p. 3:6 och  i Vägledning till ansökan p. 3:6 | x | x |
|  | 12 | Överenskommelser med uppdragsgivare/finansiär om  t.ex. anställningsförhållanden, bidrag/ersättning till prövningsplats, sjukvårdshuvudman, forskningshuvudman eller forskare. Info p. 7:2 och p. 7:3 | x | x |

**Övriga bilagor som bifogas ansökan:**
