# Supplementary material for: Effects of a mixed berry beverage on cognitive functions and cardiometabolic risk markers; A randomized cross-over study in healthy older adults
Source: PLoS One. 2017 Nov 15;12(11):e0188173. doi: 10.1371/journal.pone.0188173 (PMC5687726; doi:10.1371/journal.pone.0188173)
Supplement: S2 Text — (DOCX) [file pone.0188173.s003.docx]

# ANSÖKAN OM ETIKPRÖVNING

# Information till ansökan, *se bilaga och Vägledningar (*[*www.epn.se*](http://www.epn.se)*)*

**Till Regionala etikprövningsnämnden i:** Lund

Den regionala etikprövningsnämnd till vars upptagningsområde forskningshuvudmannen hör, se respektive nämnd *(*[*www.epn.se*](http://www.epn.se)*)*

Avgift inbetald datum:

Observera att en ansökan aldrig är komplett och därmed kan behandlas förrän blanketten är korrekt ifylld och avgiften är betald.

**Projekttitel:** Effects of food with high fiber content on the metabolism and cognitive functions

Ange en beskrivande titel på svenska för lekmän, utan sekretesskyddad information. Ange också i förekommande fall projektets identitet, projektets/forskningsplanens (protokollets eller prövningsplanens) nummer, version, datum osv.

Projektnummer/identitet:       Version nummer:

## **Uppgifter som fylls i av den regionala etikprövningsnämnden**

Ansökan komplett: Dnr:

Begäran om ytterligare information (i sak): Begärd information inkommen:

Beslutsdatum: Expeditionsdatum:

**Ansökan avser (gäller även vid begäran om rådgivande yttrande):**

Forskning där endast en forskningshuvudman deltar (5 000 kr)

Forskning där mer än en huvudman deltar (16 000 kr)

Forskning där mer än en forskningshuvudman deltar, men där samtliga

forskningspersoner eller forskningsobjekt har ett omedelbart

samband med endast en av forskningshuvudmännen (5 000 kr)

Endast behandling av personuppgifter (5 000 kr)

Forskning som gäller klinisk läkemedelsprövning (16 000 kr)

Ändring av tidigare godkänd ansökan enligt 4 § förordning (2003:615) om

etikprövning av forskning som avser människor (2 000 kr)

Om nämnden finner att forskningsprojektet inte faller inom etikprövninglagens tillämpningsområde

önskas ett rådgivande yttrande. [(Info: 4a och 4b §§ i förordning 2003:615)](http://www.epn.se/media/8604/2003_615_4ab.doc) [(Info: Bilaga till ansökan)](http://www.epn.se/media/8525/bilaga.doc)

Ja:  Nej:

**1. Information om forskningshuvudman m.m.**

**1:1 Forskningshuvudman** ([Info: p. 1:1 i Vägledning till ansökan](http://www.epn.se/media/8601/vta_p1_1.doc))

Ansökan om etikprövning av forskning ska göras av forskningshuvudmannen. *Med forskningshuvudman avses en statlig myndighet eller en fysisk eller juridisk person i vars verksamhet forskningen utförs.* Inom staten utförs forskning främst vid lärosätena, men även vid vissa andra myndigheter, som t.ex. Brottsförebyggande rådet och Socialstyrelsen. Kommuner och landsting kan vara forskningshuvudmän, liksom privaträttsliga juridiska personer.

Namn: Lunds Universitet

Adress: Getingevägen 60, Box 124, 221 00 Lund

**1:2 Behörig företrädare för forskningshuvudmannen**

Behörig företrädare är t.ex. prefekt, enhetschef, verksamhetschef. Forskningshuvudmännen bestämmer själva, genom interna arbets- och delegationsordningar eller genom fullmakt, vem som är behörig att företräda forskningshuvudmannen. Kopia av sådan handling *ska* bifogas.

Namn: Ann-Charlotte Eliasson Tjänstetitel: Prefekt

Adress: Inst. för Livsmedelsteknik, Lunds Universitet, Box 124, 221 00 Lund

**1:3 Forskare som är huvudansvarig för genomförandet av projektet (kontaktperson)**([Info: p. 1:3 i Vägledning till ansökan](http://www.epn.se/media/8714/vta_p1_3.doc))

Namn: Anne Nilsson Tjänstetitel: Teknisk doktor, forskas assistent

Adress: Enheten för Industriell Näringslära och Livsmedelskemi, Inst för Livsmedelsteknik, Lunds Universitet, Box 124, 221 00 Lund

E-postadress: anne.nilsson@appliednutrition.lth.se

Telefon: 046-2228343

Mobiltelefon: 0763 100350

**1:4 Plats** ([Info: p. 1:4 i Vägledning till ansökan](http://www.epn.se/media/8717/vta_p1_4.doc))

Plats (er) där projektet ska genomföras, ange inrättning (ar), institution (er), klinik (er) etc.

Enheten för Industriell Näringslära och Livsmedelskemi, Inst för Livsmedelsteknik, Lunds Universitet, Box 124, 221 00 Lund

**1:5 Andra medverkande**

Övriga deltagande forskningshuvudmän samt forskare ansvariga för att lokalt genomföra projektet (kontaktpersoner) anges här eller i bilaga med namn och adresser (se p. 9 bilaga nr 1).

**1:6 Ansökan/anmälan till andra myndigheter**

**Vid läkemedelsprövning**

Ansökan om tillstånd av *Läkemedelsverket* – se Läkemedelsverkets hemsida ([www.mpa.se](http://www.mpa.se/) )

Ansökan inlämnad (datum)       Tillstånd erhållits

EudraCT nr:

**Vid viss genetisk forskning**

Om personuppgifter om genetiska anlag som har framkommit efter genetisk undersökning kommer att hanteras i studien ska detta anmälas till *Datainspektionen* enligt 10 § personuppgiftsförordningen (1998:1191) – se Datainspektionens hemsida [www.datainspektionen.se](http://www.datainspektionen.se/lagar-och-regler/personuppgiftslagen/forhandskontroll/)

Anmälan inlämnad (datum)       Kommer att inlämnas efter godkänd etikprövning

**Vid viss forskning som innefattar bestrålning av forskningspersoner** ([Info: p. 9 i Vägledning till ansökan](http://www.epn.se/media/8747/vta_p9.doc))

Ansökan, enligt 16 och 22 §§ Strålsäkerhetsmyndighetens föreskrifter (SSMFS 2008:35) om allmänna skyldigheter vid medicinsk och odontologisk verksamhet med joniserande strålning, till *Strålskyddskommitté* –
för vidare information kontakta aktuell lokal strålskyddskommitté.

Ansökan inlämnad (datum):       Ansökan tillstyrkt

### 2. Uppgifter om projektet

**2:1 Sammanfattande beskrivning av forskningsprojektet (programmet)**[Vägledning till forskningsplan/forskningsprotokoll (program)](http://www.epn.se/media/8929/vägledning%20till%20forskningsplan.pdf) ([Info: p. 9 i Vägledning till ansökan](http://www.epn.se/media/8747/vta_p9.doc))

Beskrivningen ska kunna förstås av nämndens samtliga ledamöter. Undvik därför terminologi som kräver specialkunskaper. Ange bakgrund och syfte för studien samt den/de vetenskapliga frågeställning (ar) som man söker svar på. Ange de viktigaste undersökningsvariablerna. Beskriv vilka kunskapsvinster projektet kan förväntas ge och betydelsen av dessa. Ange om det är en registerstudie, uppdragsforskning etc. För fackmän avsedd detaljerad information i forskningsplan/forskningsprotokoll (program) *ska* bifogas som bilaga (se p. 9 bilaga nr 2). En utförligare beskrivning av studiens genomförande *avsedd för lekmän* kan vid behov bifogas den för fackmän avsedda obligatoriska forskningsplanen.

The metabolic syndrome is a common name for a condition involving severe risk of diabetes and cardiovascular diseases and include factors such as elevated blood sugar and insulin levels, elevated blood lipids, high blood pressure and abdominal obesity. A greater degree of chronic inflammation and increased oxidative stress is considered to be closely linked to these ohälsotillstånd. There are now strong indications that the metabolic syndrome and adult-onset diabetes also increases the risk of reduced mental (cognitive) performance. While normal aging leads to a decline in cognitive performance.

A carbohydrate-rich diet that results in a low and steady blood glucose increased (foods with low glycemic index, GI) has been shown to have a beneficial effect in the prevention and treatment of age-related diabetes, cardiovascular disease and the metabolic syndrome. A product with a high GI, on the other hand, a rapid and high elevation of glucose concentration, which has an unhealthy effect on these conditions. After ingestion of a food with a low GI usually glucose concentration sink slowly and then also come to be at a higher value at a later stage after the meal compared to a high-GI foods. This is considered to have beneficial effects on metabolism (metabolic effects), but can also hypothetically be beneficial for cognitive performance.

Some low-GI foods, such as whole grains, has been shown to have beneficial effects on blood sugar not only acute after a meal, but also for the next meal. This "second-meal effect" has been shown both from breakfast to lunch (Nilsson et al. 2008), from breakfast to dinner (Nilsson et al., 2008) and from a late dinner to breakfast the next day (Nilsson et al. 2006, 2008). The explanation for the acute hypoglycaemia after a meal is in a too-slow digestion of carbohydrates and absorption of glucose. This means that it takes longer for the glucose to enter the bloodstream with glucose increase will be lower and more prolonged. The explanation for the "second-meal effect" from breakfast to lunch, have also been explained by a more prolonged digestion and absorption resulting in prolonged lowering of free fatty acids, with a resulting increase in insulin sensitivity after taking lunch. The cause of the "second-meal effect" in the longer term, eg from breakfast to dinner or an evening meal at breakfast the following day, is not fully understood, but probably involves the mechanism behind bacterial fermentation of indigestible carbohydrates in the large intestine (colon). Indigestible carbohydrates are carbohydrates that are not degraded in and absorbed in the small intestine and continues to the large intestine and is where the nutrient substrate for bacteria. In previous studies, we have shown that eating a grain-based food can positively affect glucose tolerance and other risk markers of metabolic syndrome, such as markers of inflammation (IL-6, adiponectin) and saturation in a 10-12 hour perspective (Nilsson et al., 2006 and 2008). This improvement was correlated to increased colonic fermentation as measured by hydrogen in the breath as well as increased production of short-chain fatty acids in the colon (SCFA measured in plasma). Further, we saw in the morning after a grain-based evening meal increased plasma concentrations of the incretin hormone GLP-1 (a hormone that is released in the intestine); GLP-1 negatively correlated to blood glucose response after the breakfast meal. GLP-1 exerts numerous metabolic functions that produce effects on glucose regulation. In addition to effects on glucose control reduces the GLP-1 gastric emptying rate which can increase satiety and reduce energy intake. Due to the positive effects on glucose regulation and saturation have GLP-1 in recent years, therefore, described as an "anti-diabetic" hormone. Motion to indigestible carbohydrates are either directly or indirectly via fermentation can stimulate the release of GLP-1. Which bacteria in that case, exercising the positive effect is not known. Colonic fermentation (hydrogen content in the breath) in our previous study correlated positively to saturation and negatively to the gastric emptying rate.

Our hypothesis is that indigestible carbohydrates can have a healthy effect on the metabolism and in the prevention of metabolic syndrome and type 2 diabetes through mechanisms that are derived from the fermentation of colon bacteria of indigestible substrates, and that this effect differs depending on the choice of substrate.

It is well known that diabetes and metabolic syndrome leads to an increased risk of impaired cognitive performance. We have previously shown that the degree of glucose tolerance may influence cognitive performance even within the group of subjects with normal glucose tolerance (Nilsson et al. 2009). The brain is dependent on insulin and high insulin receptor signaling to function optimally. In insulin resistance, so that arise from metabolic syndrome and diabetes, decreasing insulin concentration and insulin receptor signaling in the brain and therefore affect the brain's cognitive capacity negatively. An improved insulin sensitivity and glucose tolerance, which has been shown, for example, after ingestion of grain-based products, might therefore have positive effects on cognitive performance.

The current project is part of a research program (Antidiabetic Food Centre, AFC). The overall objective of this research is to increase knowledge to enable the design of foods that have a positive impact on the risk factors related to obesity, age, diabetes, and cardiovascular diseases. The project for which the present application relates is to study the relationship between bacterial fermentation in the colon by indigestible substrates and systemic metabolism and cognitive performance.

Colon Substrates to be studied are:

• Products that naturally contains high content of indigestible carbohydrates, such as cereals, legumes, fruits and berries.

• Items (eg white bread) enriched with natural indigestible substrates such as dietary fiber, resistant starch and polyphenols isolated from sources containing these fermentable substrates such as cereals, legumes, fruits and berries.

The project is implemented as part studies in the Department of Nutrition and Food Chemistry, University of Lund.

a) screening for metabolic and / or cognitive effects of the test products when the product is taken in the evening, and test parameters are measured in a subsequent standardized breakfast.

b) identification of metabolic and / or cognitive power at a standardized breakfast as a test product ingested for several days (up to 1 week).

c) in the same manner as a and b, but probiotic bacteria added to the test meals (e.g., lactobacilli and bifidobacteria (bacteria added e.g. ProViva and dairy products).

That is, in total will the four studies (a, b, cb). Each sub-study contains a total of up to 4 series. The number of test products in a series is between 1-5 pc. Also included is a reference product (white bread without added fiber) in each sub-study. In every part experiment included between 17 subjects (when no cognitive parameters measured) and 40 subjects (as cognitive tests included). A "cross-over" design is used, ie each subject tested after all of the test product and the reference product (a product at a time), and each person's test results for all products are compared within the same test person. The test products are administered in a randomized order, with approximately 1 row between the two products to ensure that not there is any kvardröjd effect of a previous product.

Altogether in a substudy involved each subject in trial above the 6 time (five products + a reference product). On trial days morning 7:45 will test persons fasting from the night before (21:00) as a test product or reference product ingested. Fasting and repeated for three hours after the standard breakfast test subjects (blood test, the hydrogen in the expired air (marker for colonic fermentation), registration of subjective satiety, cognitive performance). Faeses samples provided before and after consumption of each test product for identification of bacterial flora in the colon.

**2:2 Vilken/vilka vetenskaplig (a) frågeställning (ar) ligger till grund för projektets utformning?**

Om projektet kan karakteriseras som en hypotesprövning, ange den primära och eventuellt sekundära hypotesen. Hänvisning till mer detaljerad information för fackmän kan ske till bifogad forskningsplan enligt punkt 2:1

The current project is part of a research program (Antidiabetic Food Centre, AFC). The overall objective of this research is to increase knowledge to enable the design of foods that have a positive impact on the risk factors related to obesity, age, diabetes, and cardiovascular illnesses. Increasingly data suggest that there is an interaction between the microbiota in the intestine and development of low-grade chronic inflammation, obesity and other metabolic disorders (Cani et al. 2009). Our hypothesis for the project which the present application relates is to indigestible carbohydrates can have a healthy effect on the metabolism and in the prevention of metabolic syndrome and type 2 diabetes through mechanisms that are derived from the fermentation of colon bacteria of indigestible substrates, and that this effect differs according to choice of substrate. The project for which the present application relates is to study the relationship between bacterial fermentation in the colon by indigestible substrates and systemic metabolism and cognitive performance.

**2:3 Redogör för resultat från relevanta djurförsök**

Om djurförsök inte utförts ange skälen till detta.

Animal testing is not relevant for these types of studies.

**2:4 Redogör översiktligt för undersökningsprocedur, datainsamling och datas karaktär**

([Info: p. 2:4 i Vägledning till ansökan](http://www.epn.se/media/8720/vta_p2_4.doc))

Av beskrivningen ska framgå hur projektet planeras genomföras. Beskriv insamlade datas karaktär. Ange hur datas tillförlitlighet säkerställs (t.ex. kvalitetskontroll/monitorering). - Vid enkäter och intervjuer ska beskrivas tillvägagångssätt och t.ex. frågors innehåll och hur slutsatser dras. Enkäter och skattningsskalor *ska* bifogas (se p. 9 bilaga nr 5). - För medicinsk forskning ska anges t.ex. typer av ingrepp, mätmetoder, antal besök, tidsåtgång vid varje försök, doser och administrationssätt för eventuella läkemedel och/eller isotoper, blodprovsmängd (även ackumulerad mängd vid multipla försök). Ange även om och på vilket sätt undersökningsprocedur m.m. skiljer sig från klinisk rutin. Ange proceduren för att ge den eventuella behandling efter projektets slut, som kan erfordras. Ange procedur för insamling av biologiskt material. Redogör för datakällor och procedurer vid behandling av personuppgifter. För mer detaljerad information kan hänvisning ske till bilagd forskningsplan.

The project will be implemented as part studies in the Department of Nutrition and Food Chemistry, Lund University:

a) screening for metabolic and / or cognitive effects of the test products when the product is taken in the evening, and test parameters are measured in a subsequent standardized breakfast.

b) identification of metabolic and / or cognitive power at a standardized breakfast as a test product ingested for several days (up to 1 week).

c) in the same manner as a and b, but probiotic bacteria added to the test meals (e.g., lactobacilli and bifidobacteria (bacteria added e.g. ProViva and dairy products).

The number of test products in a substudy is between 1-5 pcs. Also included is a reference product (white bread without added fiber) in each sub-study. In every part experiment included between 17 subjects (when no cognitive parameters measured) and 40 subjects (as cognitive tests included). A "cross-over" design is used, ie each subject tested after all of the test product and the reference product (a product at a time), and each person's test results for all products are compared within the same test person. The test products are administered in a randomized order, with approximately 1 row between the two products to ensure that not there is any kvardröjd effect of a previous product.

Altogether in a substudy involved each subject in trial above the 6 time (five products + a reference product). On trial days morning 7:45 will test persons fasting from the night before (21:00) as a test product or reference product ingested. Fasting and repeated for three hours after the standard breakfast test subjects.

cognitive tests

The cognitive tests that will be included in the study, including the testing of working memory (working memory, WM), attention / concentration (selective attention (SA) test), memory test for short-term memory, as well as the Stroop test. Person with extensive experience of similar tests is responsible for the cognitive tests (PhD Rade Charles Borg from the Department of Psychology, LU). WM-test and said test are similar to those described in a previous study conducted in the research (Nilsson et al. 2009).

WM test. WM is a cognitive capacity with a limited "working volume" which simultaneously can temporarily store (a few words or numbers and for a short time period) and process information. WM test thus measures the capacity to simultaneously store and process information. There are several reasons for choosing the WM as a measure of cognitive ability in the current study. WM is involved in many everyday activities such as mathematical problem solving where one should keep in mind some solution while performing further calculations. Measurements of WM has been shown to correlate to many different activities such as reading comprehension, writing protocols, follow directions, reasoning, and complex learning. Some authors (Kyllönen 1996; Engle, Kane et al. 1999) believes t o m WM and intelligence (as measured by Raven's matrices) represents virtually the same concept. While intelligence test can not be used more than once due to risk of significant learning effects are considered, however, that the cognitive test of WM can be done repeatably.

SA test: computerized test consisting of 96 images of a square divided into four smaller squares. One of the smaller squares are red and one is green and two are unstained. Images are displayed one by one in two's on the computer screen. The subjects to remember was the red and the green square are placed within the larger square. When a new image appears on the screen, the subject as quickly as possible to select, by pressing one of three buttons, the red, green, or none of the colored squares are in the same place as in the previous picture.

Dictionaries: Memory test for short-term memory (episodic memory). Dictionaries consisting of 30 words is read by two seconds apart. Immediately after the words are spoken can test subjects two minutes in which to write down as many of the words they remember. Although the test of a delayed write-off of words (10-20 min) can occur. The test is based on a well-known test "The Rey Auditory-Verbal Learning Test" (Vaisman et al. 1996).

Stroop Test: Stroop Test is designed to measure attention and ability to focus on what is essential for a long time (like the SA-test). The test is performed on the computer, and involves an interference occurs in the brain that are due to start two different cognitive processes simultaneously. The word of the colors are written in text of a different color than the text indicate. The subjects to register the color of the text and ignore the meaning of the text (what color is written). Interference occurs when due that an automatic cognitive process allows one to read a word you focus on while the controlled process of trying to decide what color the word is.

Before the trials start, the subjects have to conduct the tests so they are familiar with the tests even before the first actual trial date.

**2:5 Redogör för om insamlat biologiskt material kommer att förvaras i en biobank** ([Info: p. 2:5 i Vägledning till ansökan](http://www.epn.se/media/8723/vta_p2_5.doc))

*Med biobank avses biologiskt material från en eller flera människor som samlas och bevaras tills vidare eller för en bestämd tid och vars ursprung kan härledas till den eller de människor från vilka materialet härrör.*Redogör för var och hur prover som ska sparas förvaras, kodningsprocedurer och villkor för utlämnande av prover. Ange huvudman för biobanken. Observera att i förekommande fall ska anmälan av biobank ske till Socialstyrelsen enligt lagen (2002:297) om biobanker i hälso- och sjukvården m.m.

Collected biological material not stored in a biobank. When the study is completed and blood samples were analyzed de-identified samples. Samples were destroyed when a manuscript has been accepted for scientific publication.

**2:6 Redovisa tillgång till nödvändiga resurser under projektets genomförande**

Ange vem/vilka som har ansvaret (prefekt, verksamhetschef eller motsvarande) för forskningspersonernas säkerhet vid alla enheter/kliniker där forskningspersoner ska delta. Intyg från dessa ansvariga *ska* bifogas (se p. 9 bilaga nr 9). Av intyget ska framgå att erforderliga ekonomiska, strukturella och personella resurser finns tillgängliga för att garantera forskningspersonernas säkerhet.

See attached certificate (Car 9).

Blood samples are taken by registered nurses and experienced laboratory staff. Person with great experience in studies of cognitive tests (Associate Professor Rade Charles Borg from the Department of Psychology, LU) responsible for the tests.

**2:7 Journalföring, registrering och hantering av data** ([Info: p. 2:7 i Vägledning till ansökan](http://www.epn.se/media/8750/vta_p2_7.doc))

Redogör för hur undersökningsprocedurer och eventuella ingrepp journalförs. Ange hur registrering och behandling av resultaten ska gå till. Om materialet ska kodas, ange proceduren, vem som förvarar kodlistor och vem eller vilka som har tillgång till dem, var och hur länge de förvaras samt om materialet kommer att anonymiseras eller förstöras. Ange om band- och videoinspelningar används. Redogör för vilken tillgänglighet datamaterialet har och hur det förvaras samt hur erforderligt sekretesskydd erhålls.

When samples are coded subjects' identity with initials and the samples were coded with serial number (a number given at each time of sampling). The same applies for faecal samples. The results of the cognitive tests, encoded with the individuals first two letters of the name and surname, and the date of execution. Code lists are stored in a folder on the office responsible researcher (locked). Only the researcher and the sampler has access to the code lists. After the results are evaluated code lists will be made anonymous.

**2:8 Redogör för tidigare erfarenheter (egna och/eller andras) av den använda
proceduren, tekniken eller behandlingen**

Särskilt angeläget är att redovisning av risker för komplikationer görs tydliga och i förekommande fall med angivande av relevanta publikationer. Vid nya behandlingar av patienter, t.ex. med läkemedel, bör anges hur många patienter (med aktuell eller annan åkomma) som tidigare erhållit föreslagen behandling, läkemedelsdosering (eller annan dosering) samt hur långa behandlingsperioder som studerats.

The risks are virtually non-existent. At the Department of Applied Nutrition and Food Chemistry is a very large experience in similar studies. Many meal studies with capillary and venous blood samples were carried out by, among others that of the current project responsible scientist (eg Docket: Ethics: H4 19/2006 "Effect on glucose tolerance at the breakfast of various cereal eaten in the evening"). Similarly, there is also the experience of studies on working memory and attention in relation to glucose response (Registration no: 679/2004).

**3. Uppgifter om forskningspersoner**

**3:1 Hur görs urvalet av forskningspersoner?** ([Info: p. 3:1 i Vägledning till ansökan](http://www.epn.se/media/8726/vta_p3_1.doc))

*Med forskningsperson avses en levande människa som forskningen avser.*Ange urvalskriterier (inklusion och exklusion). Redogör för på vilket sätt forskaren kommer i kontakt med/får kännedom om lämpliga forskningspersoner. Ange om rekrytering sker från egna/andras tidigare eller pågående studier. Om annonsering sker, *ska* annonsmaterialet insändas som bilaga (se p. 9 bilaga nr 3). Om t.ex. barn eller personer som tillfälligt eller permanent inte är kapabla att ge ett eget informerat samtycke ska ingå i projektet, ska detta särskilt motiveras. Om vissa grupper (t.ex. kvinnor, barn eller äldre) utesluts från deltagande i projektet ska detta särskilt motiveras.

Healthy people between 20 and 70 years, both men and women with a BMI <30, will be included. Cognitive tests will not be included in each sub-study. When no cognitive tests included the subjects were recruited to be between 20-35 years, recruited as through advertising on campus (Lund University) (see Annex 3a) and through contact via email or telephone research people who had previously participated in similar studies and as announced they want to participate in more studies. When cognitive tests are carried out will the subjects be between 50-70 years recruited through advertisements in newspapers (see Annex 3b). The reason that an older population recruited as cognitive measurements shall be located in the issue of the project. The risk of metabolic disorders increases with age, as the risk of impaired cognitive performance. Younger subjects are more difficult to study with regard both to their current study habit (university students) and because they are still so young that their cognitive fårmåga probably not have been influenced by age and metabolism.

No public health examination requested by volunteers to participate in the studies. In the information to the subjects we inform about that participants in the study should not have any known diseases such as sturgeon metabolism. The accuracy of the research subjects are healthy are therefore based largely on the research subjects have given the correct information. They may of course have any undiagnosed illness that they do not know about themselves. If it is found that any test variables (especially sugar) is beyond the limit considered normal can research the person does not participate in the study, and the results of the analyzed samples are not used for the statistical calculations. A nurse informs the research subject and provides guidance and appropriate advice.

**3:2 Ange relationen mellan forskare/försöksledare och forskningspersonerna**

Behandlare (t.ex. läkare, psykolog, sjukgymnast) - forskningsperson (t.ex. patient, klient)

Kursgivare (lärare) - student

Arbetsgivare - anställd

Annan relation som kan tänkas medföra risk för påverkan. Beskriv: Ingen relation finns mellan forskare och forskningspersoner

3:3 Redogör för det statistiska underlaget för studiepopulationens (-ernas)/ undersökningsmaterialets (-ens) storlek [(Info: p. 3:3 i Vägledning till ansökan)](http://www.epn.se/media/8729/vta_p3_3.doc)

Redovisa statistisk styrka, så kallad ”power”- beräkning eller redovisa motsvarande överväganden som tydliggör studiens möjligheter att besvara frågeställningarna.

In a previous study in the same area where the measurement of the cognitive performance was included (Docket: 679/2004, "Effects of breakfasts with different postprandial glucose response to working and attention") was calculated that 33 subjects would be sufficient. The effect size (Cohen's d) was then estimated at d = 0:50 (which Cohen called medium effect), the two-tail test a power of 0.77 and the one-tail test a power of 0.86. A power of 80% is usually in psychological research are considered sufficient to justify an investigation. (The calculation of power based on Table 9-9 and 9-10 in the Aaron & Aaron 82003: Statistics for Psychology.) In order to design matched groups (test product, woman / man) included 40 subjects in the study, which was a number that was shown to be an adequate number. In studies where effects on cognitive functions included should therefore be included 40 subjects.

Of previous similar studies we have done, where we studied the effects of cereal based products (eg grains) on metabolic parameters and test markers of metabolic syndrome in an overnight perspective and unless measurement of cognitive functions has concluded, we have successfully used between 15-20 volunteers ( for example Nilsson et al 2006 and 2008). The number of subjects will therefore be between 15-40.Av tidigare liknande studier vi utfört där vi studerat effekter av cerealiebaserade produkter (t ex kornkärnor) på metabola parametrar och testmarkörer för metabolt syndrom i ett övernatten perspektiv och där ej mätning av kognitiva funktioner har ingått har vi med framgång använt mellan 15-20 försökspersoner (t ex Nilsson et al 2006 och 2008). Antalet försökspersoner kommer därför att vara mellan 15-40.

**3:4 Ange om forskningspersonerna kan komma att inkluderas i flera studier samtidigt
eller i annan/andra studie (-er) i nära anslutning till denna? I så fall vilken typ av forskning?** ([Info: p. 3:4 i Vägledning till ansökan](http://www.epn.se/media/8732/vta_p3_4.doc))

nej

3:5 Vilket försäkringsskydd finns för de forskningspersoner som deltar i projektet?

Det åligger forskningshuvudmannen att kontrollera att befintliga försäkringar täcker eventuella skador som kan uppkomma.

Försäkringsskydd finns i form av ett särskilt personskydd som tecknats hos Kammarkollegiet.

**3:6 Vilken ekonomisk ersättning eller andra förmåner utgår till de forskningspersoner
som deltar i projektet och när betalas ersättningen ut?** Utförligare beskrivning kan lämnas i bilaga. ([Info: p. 3:6 i Vägledning till ansökan](http://www.epn.se/media/8735/vta_p3_6.doc))

Ersättning för obehag och besvär. Belopp (före skatt): 300-500 kr per testprodukt

Ersättning för förlorad arbetsinkomst  Ja  Nej

Reseersättning  Ja  Nej

Befrielse från kostnader för läkemedel  Ja  Nej

Befrielse från andra kostnader. Vilka?

Andra förmåner. Vilka? nej

När betalas ersättningen ut? efter avslutad försöksserie. Om försökspersonen avbryter sitt deltagande i förtid får denna ersättningsbelopp som är i proportion till deltagandet.

Ingen ersättning betalas ut

### 4. Information och samtycke ([Info: Forskningspersonsinformation](http://www.epn.se/media/8598/forskningspersonsinformation.doc))

4:1 Proceduren för och innehållet i den *information* som lämnas då forskningspersoner tillfrågas om deltagande

Beskriv hur och när information ges och vad den innehåller. Ange vem som informerar. Normalt ska en kortfattad och lättförståelig skriftlig information ges. Denna skriftliga information *ska* bifogas ansökan (se p. 9 bilaga nr 4). Om ingen eller ofullständig information ges, måste skälen för detta noggrant anges.

At the first contact (project-subject) that is usually given when the subjects responds to the ad, is a brief information on the study and a general orientation is sought if the subject is suitable as a test subject (healthy and of normal weight and have the right age to attend). After the first contact sent a more detailed information by email or by letter (see Annex 4) concerning the study, the volunteers can read in peace and quiet. At the first contact we ask the subject to hear from him again if he or she remains interested in participating after reading through the information. This contact is via email or phone. The subjects may ask questions and possibly determined a time for the start of the trial dadum. Subjects were encouraged to contact the project manager, and announced that he is always welcome to contact if there is something that remains unclear.

**4:2 Hur och från vem inhämtas *samtycke*?**

Beskriv proceduren; vem som frågar, när detta sker och hur samtycket dokumenteras. Utförlig redovisning är särskilt viktig då barn eller personer med nedsatt beslutskompetens ingår i studien, likaså vid studier av en grupp/grupper, t.ex. föreningar, organisationer, företag, kyrkosamfund, församlingar eller skolklasser.

Researcher holding study sends via e-mail or letter, information relating to the study, people who got in after the announcement (see bil4). If the person decides to participate signed this information sheet at the first meeting between the subject and the experimenter / project manager. Consent signed two copies of the research person gets one and research leader second.

### 5. Forskningsetiska överväganden

5:1 Redogör för de risker som deltagandet kan medföra samt möjliga komplikationer

Dessa kan vara t.ex. fysisk skada, smärta, obehag eller integritetsintrång som projektet innebär eller kan innebära. Ange vilka åtgärder som har vidtagits för att förebygga de risker som nämns ovan samt vilken beredskap som finns för att hantera sådana komplikationer. Ange vilka/de metoder som kommer att användas för att efterforska, registrera och rapportera oönskade händelser.

If it is found that any test variables (especially sugar) is beyond the limit considered normal informs a nurse research person on this and provides guidance and appropriate advice. The risks of complications from the trials are very small. The sample results remain confidential. All results reported are based and the group averages. No single person can be identified in the reporting of results. Research subjects are healthy, participate voluntarily and are carefully informed that at any time, cancel the trials without giving any reason.

5:2 Redogör för förutsebar nytta för de forskningspersoner som ingår i projektet

The benefits that research subjects may have of the study are based on the results of the study can provide. The study gives no other specific benefit to the individual subjects.

5:3 Gör en egen värdering av förhållandet risk - nytta för de forskningspersoner som deltar

Subjects often stated that they participate because they are interested in contributing to research on nutritional health. A symbolic sum given as compensation, which for most people is too little to feel compelled to participate on economic grounds. Risks to attend the call. Research subjects contribute to important knowledge is built up. In the long term it could lead to the design of foods with health-promoting properties.

**5:4 Identifiera och precisera om etiska problem t.ex risk - nytta i ett vidare perspektiv
kan uppstå inom eller genom projektet**

Här kan redovisas om exempelvis vissa grupper kan komma att utpekas/få hjälp som ett resultat av studien.

It is not likely that there may be ethical problems from the project. The benefit of this study is that the results could advance research in karläggningen of the relationship between diet and health, possibly in an extension to affect public health. Today, a large part of the population is overweight and suffering from metabolic disorders such as diabetes type 2. It means a great suffering for the individual, but also a great cost to society. Being able to understand and then prevent / reduce this illness through diet would be a big win both for the individual and for society.

### 6. Redovisning av resultaten

**6:1 Hur garanteras forskningshuvudmannen och medverkande forskare tillgång till data (anges vid t.ex. uppdragsforskning) och vem ansvarar för databearbetning och rapportskrivning?**([Info: p. 6:1 i Vägledning till ansökan](http://www.epn.se/media/8744/vta_p6_1.doc))

Responsible for raportskrivning and data processing is tech. Dr. Anne Nilsson and prof. Inger Björck (Applied Nutrition and Food Chemistry, Lund University). Responsible for data processing in the case of the cognitive results is also Associate Professor Karl conditioned Borg (Department of Psychology, University of Lund).

6:2 Hur kommer resultaten att göras offentligt tillgängliga? Kommer studien att insändas för publicering i tidskrift eller publiceras på annat sätt?

Ange i vilken form resultaten planeras offentliggöras samt tidsplan för detta.

The results will be submitted for publication in a scientific journal.

6:3 På vilket sätt garanteras forskningspersonernas rätt till integritet när materialet offentliggörs/publiceras?

Redovisas resultat på statistisk gruppnivå? Beskriv procedurer eller metoder för avidentifiering/anonymisering.

The results are reported only on statistical group level

7. Redovisning av ekonomiska förhållanden och beroendeförhållanden

Redovisning enligt punkterna 7:1-7:3 syftar till att tydliggöra alla direkta eller indirekta förhållanden, som kan tänkas påverka forskarens relation till forskningspersonerna (vid t.ex. informations-, samtyckes-, genomförandeprocedurer).

7:1 Vid uppdragsforskning

Ange uppdragsgivaren t.ex. ett företag (vid klinisk läkemedelsprövning eller prövning av andra nya produkter), en organisation eller en myndighet.

Namn:       Kontaktperson:

Adress:       Telefon/mobiltelefon:

Ange uppdragsgivarens relation till forskningshuvudmannen/medverkande forskare, t.ex.
anställningsförhållande

**7:2 Redovisa eventuella ekonomiska överenskommelser med uppdragsgivare eller**

**andra finansiärer (namn, belopp)**

Vid klinisk läkemedelsprövning bör hänvisning ske till ingånget avtal med sjukvårdshuvudmannen. Liknande överenskommelser kan förekomma vid annan uppdragsforskning och ska redovisas på samma sätt. Separata överenskommelser med den/de som ska genomföra forskningen ska redovisas. Belopp som kommer att erhållas för studien/ersättning till kliniken/genomföraren, vad ersättningen ska täcka och ev. belopp som erhålls per forskningsperson, ska också anges här (se p. 9 bilaga nr 12).

7:3 Redovisa forskningshuvudmannens, huvudansvarig forskares och medverkande forskares egna intressen

Här redovisas t.ex. aktieinnehav, anställning, konsultuppdrag i finansierande företag, eget företag som kan få (direkt eller indirekt) ekonomisk vinst av forskningen (se p. 9 bilaga nr 12).

**8. Undertecknande**

Behörig företrädare för sökande forskningshuvudman enligt p. 1:2

Ort: Lund Datum:

Signatur: __________________________________________________________________

Namnförtydligande: Ann-Charlotte Eliasson

Tjänstetitel: Prefekt

Undertecknad forskare som genomför projektet (kontaktperson) enligt p. 1:3 intygar härmed att forskningen kommer att genomföras i enlighet med ansökan

Ort: Lund Datum:

Signatur: _________________________________________________________________

Namnförtydligande: Anne Nilsson

Tjänstetitel: Tek.dr, forskarassistent

**9. Förteckning över bilagor** ([Info: p. 9 i Vägledning till ansökan](http://www.epn.se/media/8747/vta_p9.doc))

Dokument som, i tillämpliga fall, ska bifogas *om inte motsvarande information finns i blanketten* har markerats med x. Markera de bilagor som skickas in med denna ansökan.

| **Insänd med ansökan** | **Bil nr** | Beskrivning | **Klinisk läkemedels-**  **prövning** | **Annan forskning** |
| --- | --- | --- | --- | --- |
|  | 1 | Deltagande forskningshuvudmän och medverkande forskare (kontaktpersoner) vid forskning där mer än en forskningshuvudman deltar. Info p. 1:5 | x | x |
|  | 2 | För fackmän avsedd forskningsplan, vid behov även för lekmän avsedd bilaga. Info p. 2:1 och i Vägledning till forskningsplan/forskningsprotokoll (program) | x | x |
|  | 3 | Annonsmaterial för rekrytering av forskningspersoner. Info p. 3:1 och i Vägledning till ansökan p. 3:1 | x | x |
|  | 4 | Skriftlig information till dem som tillfrågas. Info p. 4:1 och i Forskningspersonsinformation | x | x |
|  | 5 | Enkät, frågeformulär. Info p. 2:4 | x | x |
|  | 6 | Gemensam EU blankett (gäller fr.o.m. den 1 maj 2004), gäller även vid ändring. | x |  |
|  | 7 | Sammanfattning av protokollet på svenska | x |  |
|  | 8 | Prövarhandbok alt. bipacksedel/produktresumé/IB | x |  |
|  | 9 | Intyg från verksamhetschef/motsv. om resurser och om forskningspersonernas säkerhet. Info p. 2:6 | x | x |
|  | 10 | CV för forskare (samma som p. 1:3) med huvudansvar för genomförandet, redovisa forskarens (-arnas) kompetens av relevans för studien. Info i Vägledning till ansökan p. 1:3 | x | x |
|  | 11 | Beskrivning av ersättning till forskningspersoner. Info p. 3:6 och  i Vägledning till ansökan p. 3:6 | x | x |
|  | 12 | Överenskommelser med uppdragsgivare/finansiär om  t.ex. anställningsförhållanden, bidrag/ersättning till prövningsplats, sjukvårdshuvudman, forskningshuvudman eller forskare. Info p. 7:2 och p. 7:3 | x | x |

**Övriga bilagor som bifogas ansökan:**
