# Supplementary material for: Effects of a mixed berry beverage on cognitive functions and cardiometabolic risk markers; A randomized cross-over study in healthy older adults
Source: PLoS One. 2017 Nov 15;12(11):e0188173. doi: 10.1371/journal.pone.0188173 (PMC5687726; doi:10.1371/journal.pone.0188173)
Supplement: S3 Text — (DOCX) [file pone.0188173.s004.docx]

**S3 Text.** **Study protocol to experts in the ethical committee (both in Swedish and google translated into English).**

***Protocol*** *for experts (****This protocol was destined to the experts at the review board)*** *Approved by the Regional Ethical Review Board in Lund, Sweden (Dnr 2010/457 and 2011/510 (attached below)).*

Inserted below is the protocol translated with use of “Google translate”

**Protokoll för fackmän** (Protocol for experts as originally written in Swedish)

”Betydelse av kolonfermentering för metabolism och kognitiva funktioner”

### Bakgrund

Det metabola syndromet är ett samlingsnamn för ett tillstånd med allvarlig risk för diabetes och hjärt- kärlsjukdomar och inkluderar faktorer såsom förhöjda blodsocker- och insulinnivåer, förhöjda blodfetter, högt blodtryck och bukfetma [1]. En ökad grad av kronisk inflammation och en förhöjd oxidativ stress anses vara starkt knutet till dessa ohälsotillstånd [2, 3]. Det finns numera starka indikationer på att det metabola syndromet och åldersdiabetes även ökar risken för en nedsatt kognitiv prestationsförmåga [4].

En kolhydratrik kost som resulterar i en låg och jämn blodglukosstegring (livsmedel med lågt glykemiskt index, GI) har visats ha en positiv effekt vid förebyggande och behandling av åldersdiabetes, hjärt- kärlsjukdomar och det metabola syndromet [5]. Vissa låg-GI livsmedel, t ex hela kornkärnor, har visats ha gynnsamma effekter på blodsockret, inte bara akut efter en måltid, utan även efter nästkommande måltider. Denna s.k "second-meal effect" har visats både från frukost till lunch [6, 7], från frukost till middag [8] och från en sen middag till frukost dagen därpå [9, 10]. Förklaringen till den akuta blodsockersänkningen efter en måltid ligger i en förlångsammad nedbrytning av kolhydraterna och absorption av glukos. Förklaringen till "second-meal effekten" från frukost till lunch har även den förklarats av en mer utdragen matspjälkning och absorption vilket ger en förlängd sänkning av fria fettsyror, med en åtföljande höjning av insulinkänsligheten vid intag av lunchen. Orsaken till second-meal effekten i ett längre tidsperspektiv, t.ex. från frukost till middag eller från en kvällsmåltid till frukost dagen därpå, är ej helt klarlagd, men troligtvis involverar de bakomliggande mekanismerna bakteriell fermentering av odigererbara kolhydrater i tjocktarmen.

I tidigare studier har vi visat att intag av en kornbaserad måltid positivt kan påverka glukostolerans och andra riskmarkörer för metabolt syndrom, så som IL-6, adiponektin och mättnad, i ett 10-12 h perspektiv [9, 10]. Denna förbättring var korrelerad till en ökad kolonfermentering mätt med vätgas i utandningsluften samt en ökad produktion av kortkedjiga fettsyror (SCFA analyserat i plasma). Vidare såg vi på morgonen efter en kornbaserad kvällsmåltid en ökad koncentration i plasma av inkretin-hormonet GLP-1; GLP-1 korrelerade negativt till blodglukosresponsen efter frukostmåltiden. GLP-1 utövar ett flertal metabola funktioner, t ex stimulerar insulinfrisättning, ökar beta-cell massan, ökar insulinkänsligheten och inhiberar glukagonfrisättningen. Förutom effekter på glukosregleringen reducerar även GLP-1 magsäckstömningshastigheten vilket kan öka mättnadskänslan och reducera energiintaget [11]. På grund av de positiva effekterna på glukosreglering och mättnad har GLP-1 på senare år därför beskrivits som ett "anti-diabetiskt" hormon. Data tyder på att odigererbara kolhydrater antingen direkt eller indirekt via fermentering kan stimulera frisättningen av GLP-1 [12]. Kolonfermenteringen (vätgashalten i utandningsluften som markör) i vår tidigare studie korrelerade positivt till mättnad och negativt till magsäckstömningshastigheten.

Hjärnan är beroende av insulin och god insulinreceptorsignalering för att fungera optimalt. Vid insulinresistens, så som uppkommer vid metabolt syndrom och diabetes, minskar insulinkoncentrationen och insulinreceptorsignaleringen i hjärnan och påverkar därför hjärnans kognitiva kapacitet negativt. Det är välbekant att diabetes och metabolt syndrom leder till en ökad risk för nedsatt kognitiv prestationsförmåga [13, 14]. Det finns även data som tyder på att en förbättrad glukostolerans kan förbättra kognitiv prestationsförmåga [15, 16]. Vi har i tidigare studier visat att graden av glukostolerans kan påverka kognitiv prestationsförmåga även inom gruppen av personer med normal glukostolerans [17].

### Hypotes

Allt mer data tyder på att det föreligger ett samspel mellan microbiotisk aktivitet i tarmen och låggradig kronisk inflammation, fetma och andra metabola störningar [18, 19]. Vår hypotes är att fermentering i kolon av odigererbara substrat kan ha en positiv effekt på metabolismen, och att denna effekt skiljer sig beroende av val av substrat. Eftersom effekter på metabolismen, t ex glukostolerans och insulinresistens, påverkar kognitiva funktioner, är vår hypotes även att kolonfermentering av odigererbara substrat även kan påverka kognitiv prestationsförmåga.

### Syfte

Syftet med projektet för vilken den aktuella ansökan avser är att studera sambandet mellan bakteriefermentering i kolon av odigererbara substrat och systemisk metabolism samt kognitiv prestationsförmågan.

### Betydelse

Det aktuella projektet är en del i ett forskningsprogram (Antidiabetic Food Center, AFC). Den övergripande målsättningen i detta forskningsprogram är att öka kunskapen för att möjliggöra design av livsmedel som har en positiv inverkan på riskfaktorer relaterade till fetma, åldersdiabetes, och hjärt-kärl- sjukdomar. Resultatet av projektet för vilken ansökan avser kan bidra med ökad kunskap relaterad till samspel mellan kolonfermentering och riskfaktorer för metabol ohälsa samt för kognitiva funktioner. Resultatet av projektet kan också ge viktig information av betydelse för design och utveckling av hälsosamma livsmedel.

### Projektbeskrivning och metoder

*Kolonsubstrat som ska studeras är:*

- Produkter som naturligt innehåller hög halt av odigererbara kolhydrater, så som cerealier, legymer, frukt och bär.
- Produkter (t ex vitt bröd) som berikats med naturliga odigererbara substrat så som fiber, resistent stärkelse och polyfenoler, som isolerats från källor innehållande dessa fermenterbara substrat så som cerealier, legymer, frukt och bär.

*Tidigare erfarenheter av metoder, procedurer (egna och andras) särskilt med hänsyn*

*till risker samt möjliga komplikationer.*

På forskningsavdelningen där försöken ska utföras (Avdelningen för Industriell Näringslära och Livsmedelskemi, Lunds Universitet) har vi stor erfarenhet av liknande studier (ex. se ref : 7, 8, 9, 10, 14). Blodprover tas av legitimerad sjuksköterska. Person med stor erfarenhet av liknande kognitiva tester som ingår i studien ansvarar för de kognitiva testerna (Fil.dr Karl Radeborg från institutionen för psykologi, LU).

*Beskrivning av tillgång till relevant säkerhet/personal ska beskrivas.*

Mycket små risker förekommer. Produkterna som ingår i studierna är vanligt förekommande i den allmänna dieten (t ex cerealier, baljväxter eller bröd med tillsatts av kostfiber eller bär-polyfenoler (tillsatts av koncentrat av bär (t ex blåbär)). Blodprover tas av legitimerad sjuksköterska. Person med stor erfarenhet av liknande kognitiva tester som ingår i studien ansvarar för de kognitiva testerna (Fil.dr Karl Radeborg från institutionen för psykologi, LU).

*Etiska överväganden*

Om det visar sig att någon testvariabel (framför allt blodsocker) är utanför gränsen som anses normalt informerar en sjuksköterska forskningspersonen om detta, samt ger vägledning och lämpliga råd. Riskerna för komplikationer vid försöken är mycket små. Provsvaren behandlas konfidentiellt. Alla resultat baseras och redovisas på gruppmedeltal. Ingen enskild person kan därför identifieras vid resultatredovisning. Forskningspersonerna är friska, deltar frivilligt och är noga informerade om att de när som helst kan avbryta försöken utan att ange några skäl.

*Försökspersoner*

Försökspersonerna ska vara friska män och kvinnor mellan 20-70 år, BMI<30.

*Försöksdesign*

Projektet genomförs som delstudier:

a) kartläggning av metabola och/eller kognitiva effekter av testprodukter då produkten intas på kvällen och testparametrar mäts vid en efterföljande standardiserad frukost.

b) kartläggning av metabola och/eller kognitiva effekten vid en standardiserad frukost då en testprodukt intagits under flera dagar (upp till 1 vecka).

c) på samma sätt som a och b men probiotiska bakterier tillsätts testmåltiderna (t ex lactobaciller och bifidobakterier (bakterier som tillsätts t ex ProViva och mejeriprodukter).

Antalet testprodukter i en delstudie är mellan 1-5 st. Dessutom ingår en referensprodukt (vitt bröd utan tillsatt fiber) i varje delstudie. I varje delförsök ingår mellan 17 försökspersoner (då inga kognitiva parametrar mäts) och 40 försökspersoner (då kognitiva tester ingår). En "cross-over" design används, dvs varje försöksperson testas efter samtliga testprodukter och referensprodukt (en produkt i taget), och varje testpersons resultat efter samtliga produkter jämförs inom samma testperson. Testprodukterna intas i en randomiserad ordning med ca 1 v mellan två produkter för att säkerställa att inte det föreligger någon kvardröjd effekt av en tidigare produkt.

Sammanlagt i en delstudie deltar varje testperson i försök vid högst 6 tillfälle (fem produkter + en referensprodukt). På försöksdagarnas morgon 07.45 anländer testpersonerna (ca 3-4 försökspersoner/testtillfälle) fastande från kvällen innan (21.00) då en testprodukt eller referensprodukt intagits. Testmarkörer analyseras på prover som tas fastande och upprepat under tre timmar efter en standardiserad frukost som serveras ca 8.00.

*Blodprover*

Kapillärt blodprov tas för blodglukosbestämning fastande och sedan upprepat (upp till åtta gånger) under en tretimmars period. Postprandiella blodglukossvängningar, så som efter en måltid, analyseras med fördel på kapillärt blod, ej på venöst. En venflon sätts i en ven i armvecket och venösa prover tas genom denna vid samma tidpunkter som för blodglukos. Blodprover analyseras med avseende på: blodglukos, insulin, tarmhormoner (GIP, GLP-1), FFA, SCFA, antioxidativ kapacitet (så som SOD, katalas, glutationperoxidas), inflammations markörer (så som IL-6, CRP, adiponektin) och mättnadsmarkörer (så som Grelin, CCK, PYY, PP). Den sammanlagda mängden blod per försöksdag blir < 80 ml. Den sammanlagda mängden blod under ett försök blir högst 500 ml (samlat under 1,5-2 månader). Plasma och serum avskiljs efter centrifugering och placeras i frys tills de ska analyseras.

*Faecesprover*

Faecesprover kommer att tas för att kartlägga kolonflora.

*Prover på utandningsluft*

Prover på vätgas i utandningsluften (markör för kolonfermentering) tas vid samma tidpunkter som blodprovstagningen genom att försökspersonerna får andas ut ett djupt andetag i en portabel vätgasmonitor. Vätgasutsöndringen är ett mått på fermentering i tjocktarmen.

*Mättnadskänsla*

Mättnadsformulär som beskriver upplevd subjektiv mättnad, hunger och viljan att äta får fyllas i upprepat under 3 h perioden.

*Kognitiva tester*

De kognitiva testerna som kommer att ingå i studien omfattar testning av arbetsminne (Working memory, WM), uppmärksamhet/koncentrationsförmåga (selective attention (SA-test)), minnestest för korttidsminne, samt stroop test. Person med stor erfarenhet av liknande tester ansvarar för de kognitiva testerna (Fil.dr Karl Radeborg från institutionen för psykologi, LU). WM-testet och SA-testet är liknande de som beskrivs i en tidigare studie utförd i forskargruppen [17].

*WM-test*. WM är en kognitiv kapacitet med en begränsad ”arbetsvolym” som samtidigt både kan tillfälligt lagra (ett fåtal ord eller siffror och under en kort tidsperiod) och processa information. WM testet mäter således kapaciteten att samtidigt kunna lagra och processa information. Det finns flera anledningen till att välja WM som ett mått på kognitiv förmåga i den aktuella studien. WM är involverad i flera vardagliga aktiviteter som t ex matematisk problemlösning där man ska hålla i minnet en del-lösning samtidigt som man utför vidare kalkyler. Mätningar av WM har visats korrelera till många skilda aktiviteter såsom läsförståelse, protokollskrivning, följa vägbeskrivningar, resonerande, och komplicerad inlärning. Vissa författare (Kyllonen 1996; Engle, Kane et al. 1999) anser t o m att WM och intelligens (mätt med Ravens Matris) representerar så gott som samma begrepp. Medan intelligenstest ej kan användas mer än en gång pga risk för betydande inlärnings effekter anses det emellertid att kognitiva test av WM kan utföras vid upprepade mätningar.

*SA-test:* Datoriserat test bestående av 96 bilder av en kvadrat uppdelad på fyra mindre kvadrater. En av de mindre kvadraterna är röd och en är grön och två är ofärgade. Bilderna visas en och en i 2 s på dataskärmen. Försökspersonerna ska komma ihåg var den röda respektive den gröna kvadraten är placerade inom den större kvadraten. När en ny bild dyker upp på skärmen ska försökspersonen så snabbt som möjligt markera, genom att trycka på en av tre möjliga tangenter, om den röda, den gröna, eller ingen av de färgade kvadraterna är kvar på samma plats som i den föregående bilden.

*Ordlistor*: Minnestest för korttidsminne (episodiskt minne). Ordlistor bestående av 30 ord läses upp med två sek mellanrum. Omedelbart efter att orden läses upp får försökspersonerna 2 min på sig att skriva ner så många av orden de kommer ihåg. Även test av en fördröjd nerskrivning av orden (10-20 min) kan förekomma. Testet bygger på ett välkänt test ”The Rey Auditory-Verbal Learning test” [20].

*Stroop-test:* Stroop-testet är utvecklat för att mäta uppmärksamhet och förmågan att fokusera på det som är väsentligt under en längre tid (liknande SA-testet). Testet utförs på dator, och går ut på att en interferens uppstår i hjärnan som beror på att man sätter igång två olika kognitiva processer samtidigt. Ordet på färger är skrivna med text av en annan färg än vad som texten anger. Försökspersonerna ska registrera färgen på texten och ignorera textens betydelse (vilken färg som står skrivet). Interferens uppstår då p.g.a. att en automatisk kognitiv process gör att man läser ett ord man fokuserar på samtidigt som den kontrollerade processen försöker avgöra vilken färg ordet har.

Innan försöken startar ska försökspersonerna få genomföra testerna så de är bekanta med testerna redan innan det första egentliga försökstillfället.

### Referenser

1. Chew, G.T., S.K. Gan, and G.F. Watts, *Revisiting the metabolic syndrome.* Med J Aust, 2006. **185**(8): p. 445-9.

2. Paoletti, R., et al., *Metabolic syndrome, inflammation and atherosclerosis.* Vasc Health Risk Manag, 2006. **2**(2): p. 145-52.

3. Esposito, K., et al., *Inflammatory cytokine concentrations are acutely increased by hyperglycemia in humans: role of oxidative stress.* Circulation, 2002. **106**(16): p. 2067-72.

4. Gallacher, J.E., et al., *Glucoregulation has Greater Impact on Cognitive Performance than Macro-vascular Disease in Men with type 2 Diabetes: Data from the Caerphilly Study.* Eur J Epidemiol, 2005. **20**(9): p. 761-8.

5. McKeown, N.M., et al., *Carbohydrate nutrition, insulin resistance, and the prevalence of the metabolic syndrome in the Framingham Offspring Cohort.* Diabetes Care, 2004. **27**(2): p. 538-46.

6. Jenkins, D.J., et al., *Slow release dietary carbohydrate improves second meal tolerance.* American Journal of Clinical Nutrition, 1982. **35**(6): p. 1339-46.

7. Liljeberg, H.G., A.K. Åkerberg, and I.M. Bjorck, *Effect of the glycemic index and content of indigestible carbohydrates of cereal-based breakfast meals on glucose tolerance at lunch in healthy subjects.* American Journal of Clinical Nutrition, 1999. **69**(4): p. 647-55.

8. Nilsson, A.C., et al., *Effect of cereal test breakfasts differing in glycemic index and content of indigestible carbohydrates on daylong glucose tolerance in healthy subjects.* Am J Clin Nutr, 2008. **87**(3): p. 645-54.

9. Nilsson, A., et al., *Effects of GI and content of indigestible carbohydrates of cereal-based evening meals on glucose tolerance at a subsequent standardised breakfast.* Eur J Clin Nutr, 2006. **60**(9): p. 1092-9.

10. Nilsson, A.C., et al., *Including indigestible carbohydrates in the evening meal of healthy subjects improves glucose tolerance, lowers inflammatory markers, and increases satiety after a subsequent standardized breakfast.* J Nutr, 2008. **138**(4): p. 732-9.

11. Holst, J.J., *The physiology of glucagon-like peptide 1.* Physiol Rev, 2007. **87**(4): p. 1409-39.

12. Delzenne, N.M., P.D. Cani, and A.M. Neyrinck, *Modulation of glucagon-like peptide 1 and energy metabolism by inulin and oligofructose: experimental data.* J Nutr, 2007. **137**(11 Suppl): p. 2547S-2551S.

13. Awad, N., M. Gagnon, and C. Messier, *The relationship between impaired glucose tolerance, type 2 diabetes, and cognitive function.* J Clin Exp Neuropsychol, 2004. **26**(8): p. 1044-80.

14. Messier, C., *Impact of impaired glucose tolerance and type 2 diabetes on cognitive aging.* Neurobiol Aging, 2005. **26 Suppl 1**: p. 26-30.

15. Naor, M., et al., *Cognitive function in elderly non-insulin-dependent diabetic patients before and after inpatient treatment for metabolic control.* J Diabetes Complications, 1997. **11**(1): p. 40-6.

16. Meneilly, G.S., et al., *The effect of improved glycemic control on cognitive functions in the elderly patient with diabetes.* J Gerontol, 1993. **48**(4): p. M117-21.

17. Nilsson, A., K. Radeborg, and I. Bjorck, *Effects of differences in postprandial glycaemia on cognitive functions in healthy middle-aged subjects.* Eur J Clin Nutr, 2009. **63**(1): p. 113-20.

18. Cani, P.D. and N.M. Delzenne, *Interplay between obesity and associated metabolic disorders: new insights into the gut microbiota.* Curr Opin Pharmacol, 2009. **9**(6): p. 737-43.

19. Cani, P.D. and N.M. Delzenne, *The role of the gut microbiota in energy metabolism and metabolic disease.* Curr Pharm Des, 2009. **15**(13): p. 1546-58.

20. Vaisman, N., et al., *Effect of breakfast timing on the cognitive functions of elementary school students.* Arch Pediatr Adolesc Med, 1996. **150**(10): p. 1089-92.

***Förändringar i protokollet (Godkänd av den regionala etikprövningsnämnden i Lund, Sverige (Dnr 2011/510).***

”Effekter av livsmedel med högt kostfiberinnehåll på metabolism och kognitiva funktioner”, Dnr 2010/457.

Jag skulle vilja göra en ändring i den godkända ansökan. Under punkten för redogörande av undersökningsproceduren, punkt b) har vi ansökt om att testprodukt ska intas upp till en vecka.

Vi skulle här vilja ändra så att testprodukten ska intas under en period upp till 5v. Studien kommer att omfatta två testperioder á 5v då drycken jämförs med en kontrolldryck (saft). En period av 5 v kommer att skilja intaget av de olika dryckerna åt (wash-out period). Jag behöver då även ändra i forskningspersonsinformationen (bifogas). Allt annat i ansökan (Dnr 2010/457) gäller.

Vi har tidigare med fördel haft samma tidsupplägg (4-5v) då vi har undersökt effekter av omega-3 fettsyror från fiskolja (”Inverkan av skillnader i livsmedels glykemiska index samt innehåll av

antioxidanter och omega-3 fettsyror på arbetsminne och uppmärksamhet” Dnr: 100/2008).

Google translate from Swedish to English:

Protocols for experts (Approved by the Regional Ethical Review Board in Lund, Sweden (Dnr 2010/457 and 2011/510 (attached below)).

## **"Effects of colonic fermentation metabolism and cognitive functions"**

## **Effects of a mixture of berries on cardiometabolic risk markers and cognitive functions**

**Background**

The metabolic syndrome is a common name for a condition involving severe risk of diabetes and cardiovascular diseases and include factors such as elevated blood sugar and insulin levels, elevated blood lipids, high blood pressure and abdominal obesity [1]. An increased level of chronic inflammation and elevated oxidative stress is considered to be closely linked to these disorders [2, 3]. There are now strong indications that the metabolic syndrome and adult-onset diabetes also increases the risk of impaired cognitive performance [4].

A carbohydrate-rich diet that results in a low and steady blood glucose increased (food with a low glycemic index GI) has been shown to have a positive effect in the prevention and treatment of diabetes, cardiovascular diseases and metabolic syndrome [5]. Some low-GI foods, such as whole grains, has been shown to have beneficial effects on blood sugar, not only acute after a meal, but also for the next meal. This so-called "second-meal effect" has been shown both from breakfast to lunch, [6, 7], from breakfast to dinner [8] and from a late dinner to breakfast the next day [9, 10]. The explanation for the acute lowering effects after a meal is due to a slow digestion of carbohydrates and absorption of glucose. The explanation for the "second-meal effect" from breakfast to lunch, have also been explained by a more prolonged digestion and absorption resulting in prolonged lowering of free fatty acids, with a resulting increase in insulin sensitivity after taking lunch. The reason for the second-meal effect in the longer term, eg from breakfast to dinner or an evening meal at breakfast the following day, is not fully understood, but probably involves mechanisms originating from bacterial fermentation of indigestible carbohydrates in the colon.

In previous studies, we have shown that eating barley kernel based food can positively affect glucose tolerance and other risk markers of metabolic syndrome, such as IL-6, adiponectin and appetite sensations, in a perspective of 10-12 hours [9, 10]. This improvement was correlated to increased colonic fermentation as measured by hydrogen in the breath as well as increased production of short-chain fatty acids (SCFA analyzed in plasma). Further, we observes in the morning after a grain-based evening meal increased plasma concentrations of the incretin hormone GLP-1; GLP-1 negatively correlated to blood glucose response after the breakfast meal. GLP-1 exerts numerous metabolic functions, such as stimulating insulin secretion, increases beta cell mass, increases insulin sensitivity and inhibits glucagon. In addition to effects on glucose control, GLP-1 reduce gastric emptying rate which can increase satiety and reduce energy intake [11]. Due to the positive effects on glucose regulation and appetite regulation GLP-1 has in recent years been described as an "anti-diabetic" hormone. Data indicate that the indigestible carbohydrates either directly or indirectly via fermentation can stimulate the release of GLP-1 [12]. Colonic fermentation (hydrogen content in the breath as a marker) in our previous study correlated positively to satiety and negatively to the gastric emptying rate.

The brain is dependent on insulin and insulin receptor signaling to function optimally. In insulin resistance, as arise from metabolic syndrome and diabetes, decrease insulin concentration and insulin receptor signaling in the brain and therefore affect the brain's cognitive capacity negatively. It is well known that diabetes and metabolic syndrome results I an increased risk of impaired cognitive performance [13, 14]. There are also data suggesting that improved glucose tolerance may improve cognitive performance [15, 16]. We have previously shown that the degree of glucose tolerance may influence cognitive performance even within the group of subjects with normal glucose tolerance [17].

**Hypothesis**

Increasingly data suggest that there is an interaction between microbiota activity in the intestine and low-grade chronic inflammation, obesity and other metabolic disorders [18, 19]. Our hypothesis is that fermentation in the colon of indigestible substrate can have a positive effect on the metabolism, and that this effect differs depending on the choice of substrate. Since the effects on metabolism, for example, glucose tolerance and insulin resistance, affects cognitive function our hypothesis is also that colonic fermentation of indigestible substrate may affect the cognitive performance positively.

**Purpose**

The purpose of the study related to this application is to study the relationship between bacterial fermentation in the colon by indigestible substrates and systemic metabolism and cognitive performance.

**The rational and relevance**

The current project is part of a research program (Antidiabetic Food Centre, AFC). The overall objective of this research is to increase knowledge to enable the design of foods that have a positive impact on the risk factors related to obesity, type 2-iabetes, and cardiovascular diseases. The result of the project for which this application relates may contribute to increased knowledge related to the interaction between colon fermentation and risk factors for metabolic disorders and for cognitive functions. The results of the project can also provide important information relevant to the design and development of healthy foods.

**Project description and methods**

*Colon Substrates to be studied are:*

• Products that naturally contains high content of indigestible carbohydrates, such as cereals, legumes, fruits and berries.

• Products (eg white bread) enriched with natural indigestible substrates such as dietary fiber, resistant starch and polyphenols isolated from sources containing these fermentable substrates such as cereals, legumes, fruits and berries.

*Previous experience of methods, procedures (own and others) particularly with regard*

*the risks and possible complications.*

At the research department where the experiments are performed (Food for Health Science Centre (former Division of Applied Nutrition and Food Chemistry), Lund University, Sweden), we have substantial experience in similar studies (see e.g. ref: 7, 8, 9, 10, 14). Blood samples are taken by a registered nurse. Person with extensive experience in similar cognitive tests as are included in the study are responsible for the cognitive tests (PhD Karl Radeborg from the Department of Psychology, LU).

*Access to relevant safety/staff*

The study is connected with very small risks. The products included in the studies are common in the general diet (such as cereals, legumes or bread with added dietary fiber or berry polyphenols (consumed as concentrate of berries (such as blueberries)). Blood samples are taken by a registered nurse. Persons with substantial experience in similar cognitive tests which are included in the study are responsible for the cognitive tests (PhD Karl Radeborg from the Department of psychology, LU).

*Ethical considerations*

If it is found that any test variable (especially blood glucose) is beyond the limit considered as normal, the test subject will be informed by a nurse, and provides guidance and appropriate advice. The risks of complications from the trials are very small (negligible). The test results remain confidential. All results reported are based and the group averages. No single person can be identified in the reporting of results. Research subjects are healthy, participate voluntarily and is thoroughly informed that at any time they can cancel the trials without giving any reason.

*subjects*

The subjects will be healthy men and women between 20-70 years, BMI <30.

*Experimental Design*

The project is implemented as sub-studies:

a) screening for metabolic and / or cognitive effects of the test products when a product is taken in the evening, and test parameters are measured in a subsequent standardized breakfast.

b) investigations of metabolic and / or cognitive effects at a standardized breakfast when the test product has been ingested for several days (up to 1 week).

c) in the same manner as a) and b), but probiotic bacteria have been added to the test meals (e.g., lactobacilli and bifidobacteria (bacteria added e.g. ProViva and dairy products).

The number of test products in a sub-study were between 1-5. Also included is a reference product (white bread without added fiber). The sub-studies include between 17 subjects (when no cognitive parameters measured) and 40 subjects (as cognitive tests included). A "cross-over" design is used, ie each subject tested after all of the test product and the reference product (one product at each intervention), and the results for all products are compared within the same test person. The test products are administered in a randomized order, with approximately 1 week between two products to ensure that there are no remaining effects of a previous product.

In total a test subjects participate at maximum at 6 test days (five test products at maximum + a reference product). On trial days the subjects arrive at 07:45 (3-4 subjects / test day), fasting from (21:00) after a test product or reference product has been ingested. Test markers were analyzed on samples taken fasting and/or repeatedly for three hours after a standardized breakfast served about 8:00.

*Blood samples*

Capillary blood samples for blood glucose determination was taken at fasting and/or then repeatedly (up to eight times) over a three hour period. Postprandial blood glucose excursions, such as after a meal, are preferably analyzed in capillary blood. A venflon was inserted into a vein in the arm and venous samples were taken through this at the same time that blood glucose. Blood samples were analyzed for: glucose, insulin, intestinal hormones (GIP, GLP-1), FFA, SCFA, antioxidant capacity (such as SOD, catalase, glutathione peroxidase), inflammatory markers (such as IL-6, CRP, adiponectin) and satiety markers (as Grelin, CCK, PYY, PP). The total amount of blood per trial day is <80 mL. The total amount of blood in an attempt will be more than 500 ml (in total during 1.5-2 months). Plasma and serum is separated by centrifugation and placed in a freezer until analyzed.

*Faecal samples*

Faecal samples will be collected to identify colon flora.

*Samples of exhaled air*

Samples of hydrogen in the breath (marker for colonic fermentation) was analyzed at the same time points as the blood samples. For this purpose the test subjects had to exhale a deep breath in a portable hydrogen monitor. Hydrogen excretion is a measure of the fermentation activity in the large intestine.

*Satiety*

Appetite forms that described the perceived subjective satiety, hunger and desire to eat, were completed repeatedly for 3 h period.

*Cognitive tests*

The cognitive tests that will be included in the study include the testing of working memory capacity (working memory, WM), attention / concentration (selective attention (SA) test), memory test for short-term memory, as well as the Stroop test. A person with extensive experience of similar tests is responsible for the cognitive tests (PhD Karl Radeborg from the Department of Psychology, LU). The WM-test and the SA-test are similar to those described in a previous study conducted in the research [17].

*WM test:* WM is a cognitive capacity with a limited "working volume" which simultaneously can temporarily store (a few words or numbers and for a short time period) and process information. WM test thus measures the capacity to simultaneously store and process information. There are several reasons for choosing the WM as a measure of cognitive ability in the current study. WM is involved in many everyday activities such as mathematical problem solving where one should keep in mind some solution while performing further calculations. Measurements of WM has been shown to correlate to many different activities such as reading comprehension, writing protocols, follow directions, reasoning, and complex learning. Some authors (Kyllönen 1996; Engle, Kane et al. 1999) believes even that WM and intelligence (as measured by Raven's matrices) represents virtually the same concept. While intelligence test can not be used more than once due to risk of significant learning effects are considered, however, that the cognitive test of WM can be done repeatably.

*SA test*: computerized test consisting of 96 images of a square divided into four smaller squares. One of the smaller squares are red and one is green and two are unstained. Images are displayed one by one and shown in 2 seconds on the computer screen. The subjects have to remember in which position the red and the green square are placed within the larger square. When a new image appears on the screen, the subject has as quickly as possible to select, by pressing one of three buttons, if the red, green, or none of the colored squares are in the same possition as in the previous picture.

*Word lists*: Memory test for short-term memory (episodic memory). Dictionaries consisting of 30 words is read by two seconds apart. Immediately after the words are spoken can test subjects two minutes in which to write down as many of the words they remember. Although the test of a delayed write-off of words (10-20 min) can occur. The test is based on a well-known test "The Rey Auditory-Verbal Learning Test" [20].

Stroop Test: Stroop Test is designed to measure attention and ability to focus on what is essential for a long time (like the SA-test). The test is performed on the computer, and involves an interference occurs in the brain that are due to start two different cognitive processes simultaneously. The word of the colors are written in text of a different color than the text indicate. The subjects to register the color of the text and ignore the meaning of the text (what color is written). Interference occurs when due that an automatic cognitive process allows one to read a word you focus on while the controlled process of trying to decide what color the word is.

Before start of the intervention, the subjects have to conduct the tests so they are familiar with the tests even before the first actual trial date.

**References**

1. Chew, G. T., S.C. Gan, and G. F. Watts, Revisiting the metabolic syndrome. Med J Aust, 2006. 185 (8): p. 445-9.

2. Paoletti, R., et al., Metabolic syndrome, inflammation and atherosclerosis. Vasc Health Risk Manag, 2006. 2 (2): p. 145-52.

3. Esposito, K., et al., Inflammatory cytokine Increased Concentrations are acutely by hyperglycemia in Humans: Role of oxidative stress. Circulation, 2002. 106 (16): p. 2067-72.

4. Gallacher, J. E., et al., Glucoregulation has Greater Impact on Cognitive Performance than macro-vascular Disease in Men with Type 2 Diabetes: Data from the Caerphilly Study. Eur J Epidemiol, 2005. 20 (9): p. 761-8.

5. McKeown, N. M., et al., Carbohydrate nutrition, insulin resistance, and the prevalence of the metabolic syndrome in the Framingham Offspring Cohort. Diabetes Care, 2004. 27 (2): p. 538-46.

6. Jenkins, D. J., et al., Slow release dietary carbohydrate Improves second meal tolerance. American Journal of Clinical Nutrition, 1982. 35 (6): p. 1339-46.

7. liljeberg, H. G., A.K. Akerberg, and I. M. BJÖRCK, Effect of the glycemic index and content of indigestible carbohydrates of cereal-based breakfast meals on glucose tolerance at lunch in healthy subjects. American Journal of Clinical Nutrition, 1999. 69 (4): p. 647-55.

8. Nilsson, A.C., et al., Effect of Cereal test breakfasts differing in glycemic index and content of indigestible carbohydrates on daylong glucose tolerance in healthy subjects. Am J Clin Nutr, 2008. 87 (3): p. 645-54.

9. Nilsson, A., et al., Effects of GI and content of indigestible carbohydrates of Cereal-Based evening meals on Glucose Tolerance Subsequent at a standardized breakfast. Eur J Clin Nutr, 2006. 60 (9): p. 1092-9.

10. Nilsson, A.C., et al., Including indigestible carbohydrates in the evening meal of healthy subjects Improves Glucose Tolerance, Lowers inflammatory markers, and Increases satiety after a Subsequent standardized breakfast. J Nutr, 2008. 138 (4): p. 732-9.

11. Holst, J. J., The Physiology of glucagon-like peptide 1. Physiol Rev, 2007. 87 (4): p. 1409-39.

12. Delzenne, N. M., P. D. Cani, and A.M. Neyrinck, modulation of glucagon-like peptide 1 and energy metabolism by inulin and oligofructose: experimental data. J Nutr, 2007. 137 (11 Suppl): p. 2547S-2551S.

13. Awad, N., M. Gagnon, and C. Messier, The relationship between impaired glucose tolerance, type 2 diabetes, and cognitive function. J Clin Exp Neuropsychol, 2004. 26 (8): p. 1044-80.

14. Messier, C. Impact of Impaired Glucose Tolerance and Type 2 diabetes on cognitive aging. Neurobiol Aging, 2005. 26 Suppl 1: p. 26-30.

15. Naor, M., et al. Cognitive function in elderly non-insulin-dependent diabetic patient before and after inpatient treatment for metabolic control. J Diabetes Complications, 1997, 11 (1): p. 40-6.

16. Meneilly, G. S., et al., The effect of improved glycemic control on cognitive functions in the elderly patient with diabetes. J Gerontol, 1993. 48 (4): p. M117-21.

17. Nilsson, A. Rade K. Borg, and I BJÖRCK, Effects of differences in postprandial glycaemia on cognitive functions in healthy middle-aged subjects. Eur J Clin Nutr, 2009. 63 (1): p. 113-20.

18. Cani, P. D. and N.M. Delzenne, Interplay between obesity and Associated metabolic disorders: new insights into the gut microbiota. Curr Opin Pharmacol, 2009. 9 (6): p. 737-43.

19. Cani, P.D. and N.M. Delzenne, The role of the gut microbiota in energy metabolism and metabolic disease. Curr Pharm Des, 2009. 15 (13): p. 1546-58.

20. Vaisman, N., et al., Effect of breakfast timing on the cognitive functions of elementary school student. Arch Pediatr Adolesc Med, 1996. 150 (10): p. 1089-92.

Approved changes in the ethical application ((Dnr 2011/510).

To the members of the ethical committee:

***Changes in the protocol (Approved by the Regional Ethical Review Board in Lund, Sweden (Dnr 2011/510).***

We would like to make a change in the approved application. In the investigation procedure, paragraph b), we have applied for the test product to be taken up to a week. We would like to change this so that the test product will be consumed during 5 weeks. The test product will contain ordinary Swedish berries. The beverage is compared with effects of a control beverage (fruit syrup). We have previously with advantage had a similar time arrangements (4-5weeks) when we have examined the effects of omega-3 fatty acids from fish oil ( "The impact of differences in food's glycemic index and content of antioxidants and omega-3 fatty acids on working memory and attention" (Dnr, 100/2008).
